# Recent Advancement of PD-L1 Detection Technologies and Clinical Applications in the Era of Precision Cancer Therapy

**DOI:** 10.7150/jca.81899

**Published:** 2023-04-01

**Authors:** Yuanfeng Zhang, Juanjuan Wu, Chaobin Zhao, Shuyuan Zhang, Jianbo Zhu

**Affiliations:** 1 Binzhou Medical University, Yantai, Shandong, 264003, China; 2 Binzhou People's Hospital Affiliated to Shandong First Medical University, Binzhou, Shandong, 256600, China

**Keywords:** programmed death-1, programmed death-ligand 1, immunoassay, immunosuppressant, tumor, precision cancer therapy.

## Abstract

Programmed death-1 is a protein found on the surface of immune cells that can interact with its ligand, programmed death-ligand 1 (PD-L1), which is expressed on the plasma membrane, the surface of secreted cellular exosomes, in cell nuclei, or as a circulating soluble protein. This interaction can lead to immune escape in cancer patients. In clinical settings, PD-L1 plays an important role in tumor disease diagnosis, determining therapeutic effectiveness, and predicting patient prognosis. PD-L1 inhibitors are also essential components of tumor immunotherapy. Thus, the detection of PD-L1 levels is crucial, especially in the era of precision cancer therapy. In recent years, innovations have been made in traditional immunoassay methods and the development of new immunoassays for PD-L1 detection. This review aims to summarize recent research progress in tumor PD-L1 detection technology and highlight the clinical applications of PD-L1.

## 1. Introduction

Tumor immunotherapy is currently a hot research topic in the field of oncology, mainly due to the increasing incidence of tumors due to environmental changes and an aging population. Immune editing in tumors occurs in three stages: immune surveillance, immune homeostasis, and immune escape. In the immune surveillance phase, effective antigen delivery and T cell activation enable effector T cells to effectively clear tumor antigens, which initiate an immune response to inhibit tumor development. Tumor cells surviving in the immunosurveillance phase can enter the equilibrium phase in which tumor cells mutate and induce immune suppression to avoid continuous immune stress and keep themselves in a functional state. In the escape phase, various immunosuppressive molecules and cytokines are activated by tumor cells and promote tumor growth, leading to immune escape and disease progression 1.

Programmed death-ligand 1 (PD-L1), encoded by the CD274 gene, is a receptor expressed by tumor cells and their associated stromal cells. PD-L1 is the ligand for programmed death-1 (PD-1), which is found on the surface of tumor-killing T cells. This interaction can result in the apoptosis of activated T cells and the loss of the ability to recognize tumor cells, which might increase the spread of cancerous metastases. Additionally, it has been shown that PD-L1 can be influenced by the tumor suppressor gene P53 through miR-34 and enhance the epithelial-mesenchymal transition (EMT), which is what causes cancer to metastasize 2,3,4. The overexpression of PD-L1 in cancers such as gastric carcinoma, hepatocellular carcinoma, renal-cell carcinoma, esophageal carcinoma, pancreatic cancer, ovarian cancer, and bladder cancers is associated with poor clinical outcomes, and the detection of its levels has important clinical significance 1. This review provides a summary of the clinical applications of PD-L1 and the current status of PD-L1 assays, aiming to provide a comprehensive picture of current PD-1/PD-L1 research and provide directions for future treatment strategies.

## 2. Mechanism of PD-L1 action

**Figure 1:**
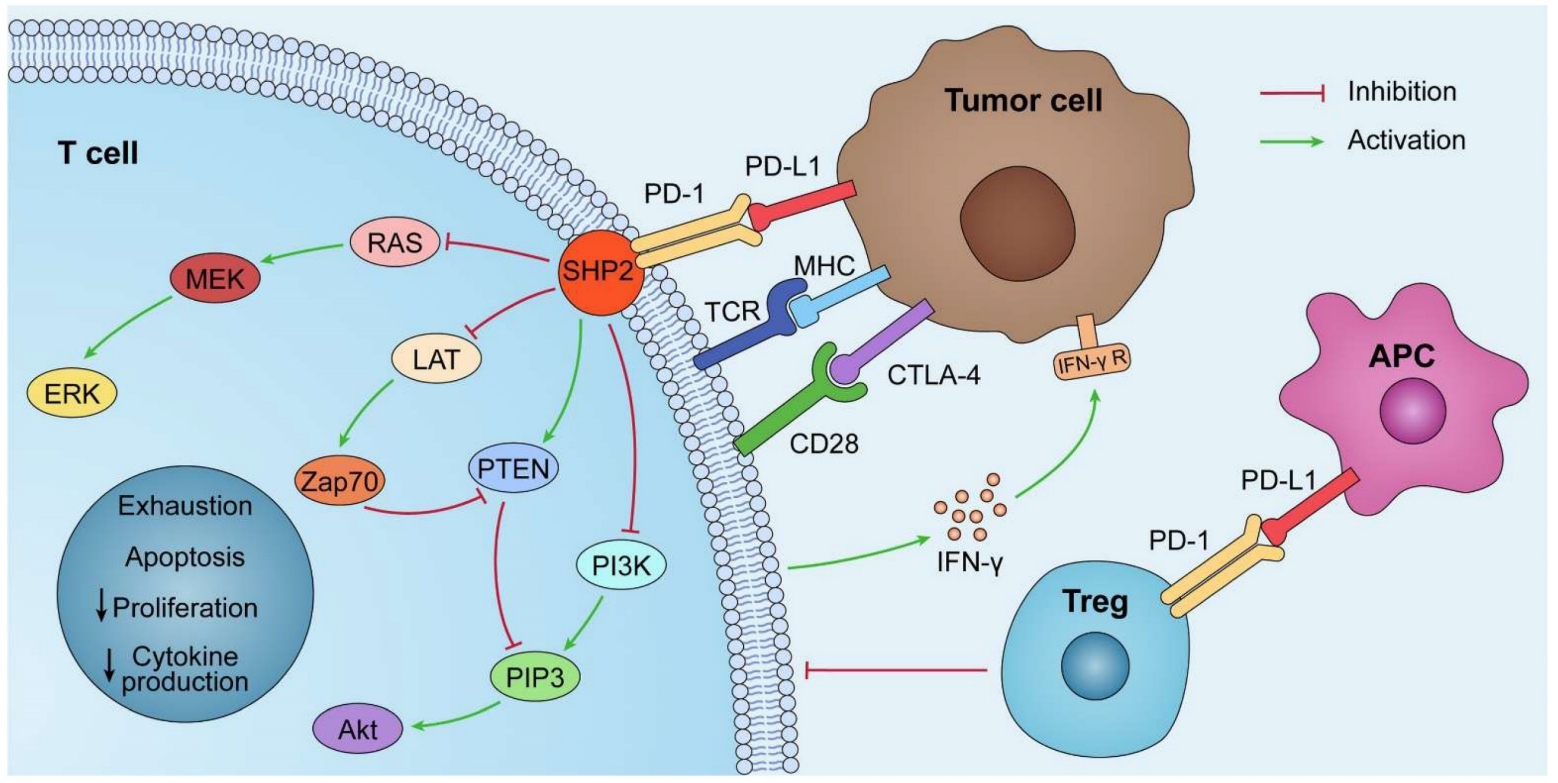
** The PD-1/PD-L1 pathway plays a role in immunosuppression.** PD-L1 is abnormally expressed on tumor cells and APCs in the tumor microenvironment. When PD-L1 interact with PD-1, PD-1 can recruit tyrosine phosphatase SHP2, inactivating CD28 and T cell receptor (TCR) function and signaling pathway: PI3K/PIP3/Akt or RAS/MEK/ERK, which decreases CD8^+^ T cell proliferation, survival, and cytokine production.

By interacting with PD-1 receptors on the surface of T cells, PD-L1 on the surface of tumor cells can induce the death of activated T cells and boost IL-10 production in human peripheral blood, thereby promoting immunosuppression and tumor progression 5. The PD-1/PD-L1 axis was discovered to play a role in the generation and function of Tregs, and the induction of T cell Foxp3 expression *in vitro* or *in vivo* can result in Treg-like immunosuppression. PD-L1 enhances the expression of Foxp3 in Tregs, thus boosting their immunosuppressive potential. PD-L1 can further supplement this immunosuppressive ability by simultaneously upregulating PTEN and downregulating Akt, mTOR, and ERK2 to transform naive CD4^+^ T cells into Tregs 6,7** (Fig. 1)**.

PD-L1 is also crucial for intestinal mucosal tolerance. Reiynoso et al. identified the importance of gut mucosal PD-L1 in a steady-state tolerogenic response. Experiments in an iFABP-tOVA transgenic mouse model—which is used to study the role of PD-L1 in CD8^+^ T cell tolerance to an intestinal epithelium-specific Ag—showed that inhibiting PD-1/PD-L1 signaling altered intestinal tolerance to autoantigens and induced CD8^+^ T cell-mediated autoimmune enteritis 8. PD-1/PD-L1 signaling also plays a significant role in the generation of cells that can produce the inflammatory cytokines IFN-g and IL-17A. Chronic inflammatory illnesses of the bowel, such as Crohn's disease, ulcerative colitis, and celiac disease, and chronic infections, such as *Helicobacter pylori,* have been associated with aberrant PD-L1 expression and/or signal transduction 9.

PD-L1 lacks a traditional signaling motif, but it contains unconventional motifs that play a role in IFN-γ-mediated cytotoxicity resistance 10. It regulates the EMT process, which is the transition of epithelial cells into mesenchymal-like cells with migratory and invasive activities. In colorectal cancer, PD-L1 was shown to enhance EMT through the RAS/MEK/ERK pathway and to interact with the 1-86 amino acid segment of KRAS and transduce signals 11. In contrast, in non-small cell lung cancer (NSCLC), overexpressed PD-L1 enhances the migration and invasion of cancer cells by activating the PI3K/AKT pathway 12. In addition, PD-L1 can directly bind to integrin 4 to activate the AKT/GSK3 signaling cascade and then stimulate the transcriptional repressor SNAI1, which decreases SIRT3 promoter activity, hence promoting EMT and increasing glucose uptake of tumor cells** (Fig. 1)**. Apart from glucose uptake, PD-L1 promotes lipid uptake in tumor cells by elevating the expression of fatty acid-binding proteins in gastric adenocarcinoma, hence evading tumor immunity 13. It has also been demonstrated that PD-L1 plays a crucial function in maintaining the stemness of tumor cells and regulating autophagy 14,15,16.

## 3. Clinical applications of PD-L1

### 3.1. PD-L1 predicts prognosis and guides treatment

PD-L1 is an excellent biomarker for predicting prognosis and therapeutic response, thereby guiding the choice of treatment modality and improving precision medicine 17. For example, PD-L1 overexpression in hepatocellular cancer, pancreatic cancer, renal cell carcinoma, ovarian cancer, and bladder cancer correlates with poor clinical outcomes; however, the prognostic value of PD-L1 is not absolute and depends on a variety of factors. Tessier-Cloutier et al. used immunohistochemistry (IHC) to examine the prevalence of PD-L1 expression in pancreatic ductal adenocarcinoma and compared it with clinical characteristics, including MMR status and tumor-infiltrating lymphocytes (TILs), to determine if there was a correlation with clinical outcomes. As such, increased PD-L1 expression was associated with a worse outcome (p = 0.0367); however, there was no statistically significant correlation between PD-L1 status and MMR status or TILs 18. For hepatocellular carcinoma, Chang et al. assessed the levels of soluble circulating PD-1 (sPD-1) and PD-L1 (sPD-L1), as well as membrane-bound PD-L1. The data suggest that sPD-1 and sPD-L1 are separate prognostic variables that play opposing roles in predicting both disease-free survival and overall survival (OS) in hepatocellular carcinoma patients 19. In contrast, most studies have reported that PD-L1 expression is associated with better clinical outcomes in breast cancer and Merkel cell carcinoma. Baptista et al. performed an immunohistochemical study of cancer tissue from 192 cases of stage I, II, and III breast cancer. They found that PD-L1 expression reached 56.6% (107/189) and that its expression was significantly associated with better OS (p = 0.04) in breast cancer patients 20. Schalper et al. studied YTMA128 (N = 238) and YTMA201 (N = 398), a retrospective collection of stage I-III breast cancers from Yale University. They found that 55.7% and 59.5% of cases showed PD-L1 mRNA expression, respectively, and that higher expression was significantly associated with increased TILs (P = 0.04) and longer relapse-free survival (P = 0.01) 21.

Despite these promising results, the prognostic value of PD-L1 expression remains controversial in lung cancer, colorectal cancer, and melanoma. In the case of colorectal cancer, there are notable variations in the outcomes of various studies. Huang et al. reported that patients with high CD8^+^ TILs/tumor-positive PD-L1 have a better prognosis in stage II-III colon cancer 22. Wyss et al. discovered that stromal PD-L1 expression was related to a better prognosis, resulting in a longer OS and disease-free survival. Moreover, PD-L1 labeling in the tumor cells was less frequent than stromal staining and did not affect the outcome 23. On the other hand, Jiang et al. reported that high PD-L1 expression is an independent predictor of low postoperative disease-free survival in colorectal cancer patients with a high TLG3.0 24. In addition, the expression of PD-L1 in the tumor microenvironment showed prognostic value for melanoma, NSCLC, and bladder cancer, but not in every study. Notably, in some tumors, such as bladder cancer, PD-L1 expression on immune-infiltrating cells rather than tumor cells shows predictive value, but for NSCLC, PD-L1 expression on both immune and tumor cells exhibits predictive value 25.

In clinical practice, PD-L1 detection is mostly based on primary tumors. Few studies have examined the differences in PD-L1 positivity between different metastases and primary tumors, but this variability in expression is worth exploring. By comprehending the patterns of PD-L1 expression in primary and metastatic cancer foci, it is possible to enhance our understanding of the tumor microenvironment at different locations and improve biopsy techniques. In addition, PD-L1 content as a potential biomarker for predicting the response to anti-PD-L1 therapy may also require the analysis of metastatic lesions 26,27. Zhang et al. evaluated the differential expression of PD-1, PD-L1, and PD-L2 between primary and metastatic sites of renal cell carcinoma. In the entire cohort (N = 163), the IHC results showed that the rate of PD-L1 detection was consistently low at 32.5%, but it was detected more frequently in metastatic sites than in the primary tumor site (χ2 = 4.66, p = 0.03). PD-L1 had high expression in the lung/lymph nodes (37.5%) but low expression in bone metastases (12.2%). Thus, anti-PD-L1 treatment outcomes may correlate with different metastatic sites, and this precise detection is an effective way to achieve individualized treatment 28. Similarly, Moutafi et al. observed higher PD-L1 expression in metastatic lung cancer samples compared to primary tumor samples. Specifically, 33.8% of metastatic lung cancer samples showed high PD-L1 expression (tumor proportion score ≥ 50%), while only 28.4% of primary tumor samples showed the same level of PD-L1 expression 29** (Table 1)**. In conclusion, PD-1/PD-L1 expression is associated with histologic type and varies between primary and metastatic tumor sites. Thus, this review could have an impact on patient management and increase awareness of these factors for future clinical trial designs.

### 3.2. Anti-PD-L1 monoclonal antibodies

With the success of PD-1/PD-L1 axis blocking trials in solid tumors, immunotherapy has assumed a central position in the treatment of cancer, and many clinical trials with anti-PD-L1 monoclonal antibodies (mAbs) have been developed. Currently, the U.S. Food and Drug Administration has approved anti-PD-L1 mAbs, including Pembrolizumab, Nivolumab, Durvalumab, Avelumab, and Atezolizumab, as PD-1/PD-L1 inhibitors 31. PD-1/PD-L1 inhibitors have shown very good results in treating patients with some solid tumors, including melanoma, lung cancer, kidney cancer, and head and neck cancer, where prolonged OS was seen in patients 32. The 5-year survival rate of NSCLC patients treated with PD-1/PD-L1 inhibitors increased from 5% during the period of chemotherapy to 16-23%, showing outstanding efficacy 33. PD-1/PD-L1 inhibitors have been the most extensively studied and used in the treatment of melanoma. Nivolumab has demonstrated efficacy in the treatment of squamous cell lung cancer and Hodgkin's lymphoma, while MPDL-3280A, an anti-PD-L1 mAb, is effective in the treatment of bladder cancer and NSCLC 34.

However, the efficacy of PD-1/PD-L1 inhibitors has only been demonstrated in a limited number of tumor types, with less than 30% of patients exhibiting robust treatment responses 35. The Keynote-028 clinical study examined Pabolizumab therapy in 26 individuals with recurrent glioblastoma and found it to be ineffective. Moreover, multiple investigations of high-grade glioblastoma (WHO Grades 3 and 4) have demonstrated the limited efficacy of Pabolizumab treatment relative to a placebo 36,37. Several studies have reported that PD-1/PD-L1 inhibitor therapy may promote hyperprogressive disease, which is an unexpected acceleration of tumor growth and progression. This typically results in a significant decrease in survival time and has been observed in a variety of tumor types 31. Unfortunately, the underlying mechanisms and features of hyperprogressive disease in the setting of immunosuppressive medication remain poorly understood. In clinical practice, most patients develop more severe drug resistance over time, even though some patients may achieve long-term effectiveness. The frequent development of medication resistance, whether acquired or primary, remains a significant issue that restricts its therapeutic use and is becoming a growing concern in the field 38.

Patient responses to PD-1/PD-L1 blockade are not identical, and patients can be classified as responders, non-responders, and those who develop resistance based on their responses to anti-PD-1/PD-L1 antibodies 39. As stated above, although anti-PD-1/PD-L1 antibodies exhibit potent antitumor effects in some patients, most patients do not benefit from PD-1/PD-L1 therapy due to primary or acquired treatment resistance 35,40. However, in addition to PD-L1 signaling, negative factors, such as immunosuppressive cells, cytokines, cancer-associated adipocytes, overactive cancer-associated fibroblasts, other immune checkpoints, and aberrant angiogenesis, can all influence cancer-immune set points and create an environment of immune tolerance. On the other hand, some positive factors, such as immunogenic cancer cell death, immune-supporting cytokines, and professional antigen-presenting cells, can contribute to immune clearance 41,42,43. Therefore, removing these negative factors may enhance the therapeutic effect of anti-PD-1/PD-L1 antibodies and reduce drug resistance. Correspondingly, enhancing these positive factors may promote tumor immune response.

**Figure 2:**
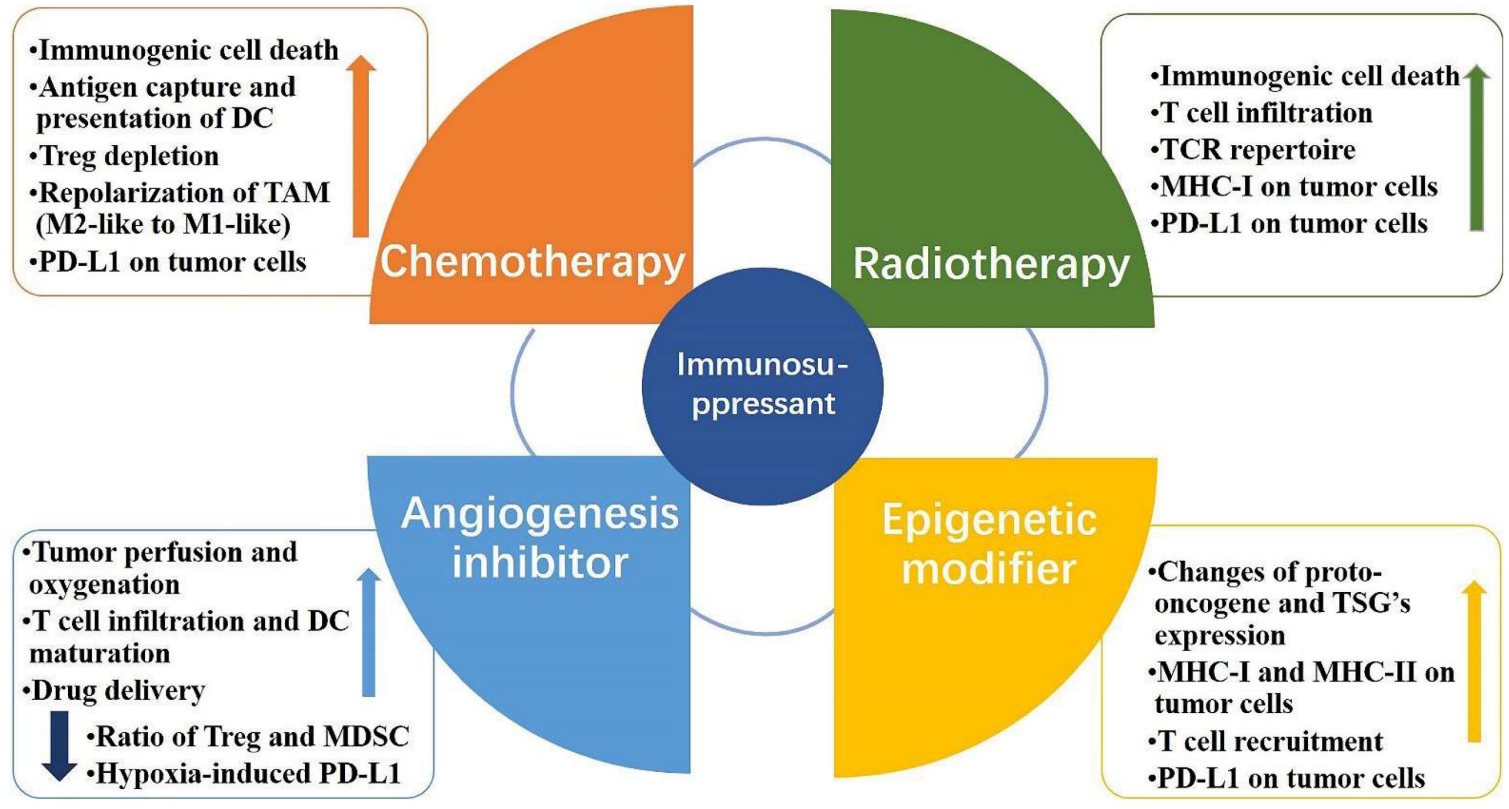
** Mechanism of PD-L1 immunosuppressants combination therapy.** Combination therapeutic strategies of PD-L1 immunosuppressants can enhance the body's innate and adaptive immunity, induce immune clearance, and attenuate immunosuppression. The increase in PD-L1 on tumor cells brought about by chemotherapy, radiotherapy, and epigenetic modifiers can be effectively slowed or eliminated by PD-L1 immunosuppressants; PD-L1 immunosuppressants combined with angiogenesis inhibitors can directly reduce hypoxia-induced PD-L1 and attenuate immune escape.

Combining anti-PD-1/PD-L1 antibodies with other therapeutic approaches can help counteract the negative factors of tumor immunity while boosting the positive factors and improving the response rate of patients. Anti-PD-1/PD-L1 antibodies in combination with conventional approaches, such as radiotherapy and chemotherapy, play an important role in the treatment of clinical malignancies. Recently, combination therapies have shown significant effects in preclinical models and clinical trials. Radiotherapy combined with anti-PD-1/PD-L1 antibodies exerts a combined effect through activation and inhibition. On the one hand, the positive effect of radiotherapy on immunomodulation can be exploited to increase the sensitivity of tumor cells to anti-PD-1/PD-L1 antibodies. On the other hand, radiotherapy may upregulate PD-L1 expression in tumor tissues and exert a negative immunomodulatory effect, which can be counteracted by anti-PD-1/PD-L1 antibodies through their pathways of action 44,45 **(Fig. 2)**. The CheckMate 816 phase III trial recently confirmed the benefit of using a combination approach. In this trial, three cycles of Nivolumab combined with platinum-based two-drug chemotherapy resulted in a significantly higher incidence of pCR (24% vs. 2.2%) and a higher MPR rate (36.9% vs. 8.9%) compared to chemotherapy alone 46. Altorki et al. compared neoadjuvant Durvalumab alone or in combination with stereotactic radiotherapy for patients with early-stage NSCLC. The difference in the major pathological response rates between the groups was significant. A major pathological response was observed in 2 (6.7% [95% CI: 0.8-22.1]) of 30 patients in the Durvalumab monotherapy group and 16 (53.3% [95% CI: 34.3-71.7]) of 30 patients in the Durvalumab plus radiotherapy group 47. In addition, novel therapeutic agents of CTLA4 inhibitors, angiogenesis inhibitors, and epigenetic modifiers have also been combined with anti-PD-L1 antibodies to play an important role in oncology treatment 48,49,50 **(Table 2)**. Clinical trials of combination therapies involving anti-PD-L1 mAbs are far more common than monotherapy trials, accounting for more than 80% of the total, and VEGF/VEGFR-targeted therapy, chemotherapy, and CTLA4 inhibitors remain the preferred strategies for combination therapy 51.

### 3.3. PD-L1 small molecule inhibitors

PD-L1 small molecule inhibitors can overcome the limits of anti-PD-L1 mAbs, including poor tissue and tumor permeability, long half-life, and poor oral bioavailability, which has motivated researchers to focus on peptide and non-peptide small molecule inhibitors as viable substitutes or supplements to anti-PD-L1 mAbs 55.

PD-L1 small molecule inhibitors achieve tumor immunotherapy in three main ways: 1) blocking the direct interaction between PD-1 and PD-L1, 2) inhibiting PD-L1 transcription and translation, and 3) promoting the degradation of the PD-L1 protein 56. In 2014, the first peptide-based inhibitor of human PD-1, called AUNP-12, was reported and was jointly developed by Aurigene and Pierre Fabre's laboratory in India 57. In recent years, D-peptide antagonists, macrocyclic peptides by Bristol-Myers Squibb (BMS), and non-peptide small molecule inhibitor derivatives from the BMS series (e.g., A22, PDI-1, and P-18) have also been identified 58. A large number of patents on small molecule inhibitors of PD-1/PD-L1 have emerged, yet most studies are still in preclinical stages, with only a few reaching clinical trials **(Table 3)**. Currently, only INCB086550, a novel oral small molecule PD-L1 inhibitor, has had its phase I study results reported, indicating that the development of marketable small molecule PD-1/PD-L1 targeted therapeutics is a challenging process 59,60. One of the difficulties is creating drugs with a strong affinity for targets that lack stable or pocket-like active sites. As neither PD-L1 nor PD-1 has a deep binding pocket, their interaction occurs at a hydrophobic, flat, and extended (~1.700 A) interface. Another challenge for small molecule immuno-oncology drugs is that many immunotherapeutic targets and pathways are interconnected, meaning that modulation of one target may affect other immune signaling pathways 61. As such, a growing number of studies are focusing on addressing these challenges. Lin et al. summarized the structural requirements for designing PD-1/PD-L1 small molecule inhibitors, pointing out that hydrophobic interactions, aromatic ring systems, amide groups, and heterocycles are key structural features common to anti-PD-L1 compounds 62. Most of the current studies on the construction of PD-L1 small molecule inhibitors have focused on computerized docking model structures based on PD-1/PD-L1 molecular docking and the synthesis of the original BMS compounds, such as BMS-202 and 1166-related derivatives. Subsequently, several studies have reported conventional and routine computerized screening methods for small compound libraries. Akiyama et al. found six compounds following three rounds of screening among 67,395 compounds from the Shizuoka small compound library. SCL-1 and SCL-2 exhibited moderate inhibitory activity against PD-1/PD-L1 binding compared to anti-PD-1 antibodies, with SCL-1 showing significantly weaker cytotoxic effects on the target cells than the other compounds 58. Due to the limited number of small molecule PD-1/PD-L1 inhibitors available, there is an opportunity to explore novel antagonists that offer optimal therapeutic efficacy; thus, it is likely that PD-L1 small molecule inhibitors will have a promising future.

### 3.4. Cancer vaccines with PD-L1 blockade

Cancer vaccines help the immune system recognize and remember the antigens expressed by cancer cells, thus effectively launching a response against the tumor and eliminating it. To date, the number of preventive vaccines has exceeded the number of therapeutic vaccines 63. There is a wide variety of cancer vaccines, including cellular vaccines, nucleic acid vaccines, protein-peptide vaccines, and genetically enfgineered vaccines 64. Currently, the most popular cancer vaccine regimens first require the discovery and identification of tumor cell-associated antigens, and almost half of the clinical trials underway involve one or more antigens. Neoantigens are tumor cell-specific proteins that are ideal cancer-specific targets, and neoantigen-based cancer vaccines have shown outstanding therapeutic efficacy 65. In addition, the ability of dendritic cells (DCs) to uptake, process, and present antigens makes them key modulators of adaptive immune responses, and DC-based cancer vaccines occupy a dominant position in the field. Although only a few neoantigen-based DC vaccines have been studied in clinical trials, they have a vast scope for exploration 66. However, vaccinations against established malignancies have mostly shown limited clinical benefits. The efficacy of tumor-specific antigen vaccines may be influenced by immune checkpoint suppression mechanisms, such as PD-1/PD-L1 attenuating antigen presentation and impeding T cell-mediated cytotoxicity 67. In addition, in terms of checkpoint blockade therapy, many patients cannot receive such treatment due to weak anti-cancer immunity. Therefore, there is a need to increase the frequency and function of CD8^+^ T cells in patients by targeting immunogenic and tumor-restricted antigens (e.g., neoantigens) using effective vaccination platforms 68. Recently, studies have shown that a therapeutic strategy of PD-1/PD-L1 blockade combined with cancer vaccines is safe and effective. In June 2022, Guo et al. reported the first study of complete and durable regression of gastric tumors achieved by combination therapy with a neoantigen-based DC vaccine and an immune checkpoint inhibitor. A personalized neoantigen-loaded monocyte-derived DC (Neo-MoDC) vaccine was administered alone to a patient with advanced metastatic gastric cancer for the first two months, followed by combination therapy with Nivolumab. The Neo-MoDC vaccine was found to induce neoantigen-specific CD4^+^ T cell and CD8^+^ T cell activation and a significant increase in the frequency of peripheral blood neoantigen-specific T cell clones. Tumor size decreased rapidly after the start of combination therapy and lasted for more than 25 months 69.

There are two main strategies for the combination of PD-1/PD-L1 blockade and cancer vaccines: the preparation of chimeric vaccines, which modify cancer vaccines to have anti-PD-1/PD-L1 effects; and drug combinations, where PD-1/PD-L1 blockers are directly combined with cancer vaccines for tumor treatment. Pan et al. prepared a CTLA-4-PD-L1 chimeric protein vaccine and found that rats carrying intrahepatic cholangiocarcinoma had increased PD-L1 and CTLA-4 antibody titers and reduced the intrahepatic cholangiocarcinoma tumor load after receiving the vaccine 70. Huang et al. developed a novel adeno-associated virus neoantigen vaccine modified with Toll-like receptor 9 inhibitory fragments, PD-1 trap, and PD-L1 miRNA to overcome PD-1/PD-L1 inhibition in DCs. The results showed that these functional PD-1 traps and PD-L1 miRNAs overcame the PD-1/PD-L1 inhibition mechanism and improved the efficacy of radiotherapy 71. Bai et al. directly combined PD-1/PD-L1 blockers and cancer vaccines by administering a bio-nanoparticle-based λ phage vaccine—targeting aspartate beta-hydroxylase alone or in combination with PD-1 blockers—to mice bearing syngeneic hepatocellular carcinoma or triple-negative breast cancer tumors. A combination therapy (vaccine + PD-1 inhibitor) was found to significantly inhibit the growth of primary liver and mammary tumors 72. Overall, while many cancer vaccines offer the opportunity to prevent and treat cancer, they have struggled to show improved OS and progression-free survival (PFS). PD-1/PD-L1 blockers in combination with cancer vaccines have shown promising findings; however, there is currently little clinical application, and more in-depth studies of this combination strategy are urgently needed to improve its anti-cancer efficacy.

## 4. Detection methods

Traditional immunoassay approaches, such as IHC, enzyme-linked immunosorbent assay (ELISA), and immunofluorescence (IF), which have experienced some innovations in recent years, are primarily utilized for the detection of PD-L1. New PD-L1 immunoassays, including electrochemical immunoassay, photochemical immunoassay, and nuclear medicine imaging, have been used for the quantitative detection of PD-L1 due to their high sensitivity, low detection limit, and broad detection range; however, relatively few reports are available. The following is an overview of the published literature on the topic of novel immunoassays, including electrochemical immunoassays, photochemical immunoassays, and nuclear medicine imaging, as well as innovative perspectives on standard immunoassays, including IHC, ELISA, and IF.

### 4.1. Conventional immunoassay

The traditional methods for PD-L1 detection include IHC, ELISA, flow cytometry, and IF. Among them, IHC is the most widely used in the membrane PD-L1 assay because of its high efficiency and rapidity, while ELISA occupies the main position in the sPD-L1 assay as a stable and convenient assay.

#### 4.1.1. Immunohistochemistry

PD-L1 is the only diagnostic marker now licensed for use in clinical practice, and IHC has been widely employed for PD-L1 detection due to its efficiency and speed. To date, four standardized PD-L1 IHC assays have been approved for clinical use by the U.S. Food and Drug Administration, including 22C3 and 28-8 PharmDx on the Dako platform, SP142 and SP263 on the Ventana platform. In addition, Cell Signaling Technology's clone E1L3N is also one of the most widely used and least expensive PD-L1 antibodies 73. IHC detection of PD-L1 is achieved through multiple technologies and platforms that are not fully equivalent. The differences in antibody clones, IHC platforms, detection systems, and scoring algorithms can all have an impact on detection, causing PD-L1 IHC assay results to vary widely 74. As such, research has focused on the consistency of PD-L1 detection by different mAbs. Scheel et al. conducted an experiment in 2016 to investigate interobserver concordance and PD-L1 IHC staining patterns using 15 lung carcinoma resection specimens (n = 11 for adenocarcinomas and n = 4 for squamous cell carcinomas). The samples were centrally stained with assays 28-8, 22C3, SP142, and SP263. It was found that SP142 stained fewer cancer cells compared to 28-8, 22C3, and SP263 in four lung cancer specimens, while SP263 stained more cancer cells in nine specimens 75. However, this study had a relatively small sample size. Subsequently, the Blueprint Project evaluated the same four antibodies in 2017 with 39 NSCLC specimens, demonstrating comparable percentages of stained tumor cells for 22C3, 28-8, and SP263, while SP142 showed fewer stained tumor cells overall 76. In the same year, Ratcliff et al. evaluated the degree of concordance between Ventana SP263 (Durvalumab), Dako 22C3 (Pembrolizumab), and Dako 28-8 (Nivolumab). Consistent with the findings of the Blueprint Project, the three assays exhibited similar tumor staining patterns in 493 NSCLC samples, achieving an overall percentage agreement of more than 90% 77.

It is important to note that IHC testing has some limitations, including the potential for tumor false positives. This may be due to PD-L1-positive lymphocytes and histiocytes being interspersed with PD-L1-negative tumor cells, which can lead to negative tumor cells being stained as positive. Additionally, granular cytoplasmic staining of non-membranous malignancies may be mistakenly interpreted as positive 74. Another limitation is that tumor heterogeneity cannot be avoided. The expression levels of PD-L1 may vary greatly between various tumor regions, and the quality of tumor tissue collection is a significant determinant of PD-L1 identification by IHC. The immune activation response of tumor cells at the interface between the tumor and stroma may result in preferential expression of PD-L1 in this area. However, tiny tumor biopsies may miss the critical tumor-stromal interface. Ilie et al. observed a concordance of only 48% between resected and biopsied PD-L1 levels 78. Further limitations include the fact that dynamic, real-time detection of PD-L1 content *in vivo* is not possible, and there is also controversy surrounding the use of archived vs. fresh specimens for testing. Takeda et al. evaluated the expression of PD-L1 in archived and fresh samples and found that it was significantly lower in archived specimens 79. However, the KEYNOTE-010 trial 80 and the FIR study 81 demonstrated that either fresh or archived tissues could be reliably assessed for PD-L1 status by IHC.

#### 4.1.2. Enzyme-linked immunosorbent assay

ELISA is primarily subdivided into indirect assay, double antibody sandwich assay, and competitive binding assay. Currently, based on the easy access and non-invasive characteristics of blood samples, ELISA is frequently utilized for the detection of sPD-L1 82.

In recent years, with in-depth research on sPD-L1 and the innovation of its antibody synthesis, a lot of progress has been made in the double antibody sandwich ELISA method. Huang et al. enhanced the classic ELISA method by employing a novel Aptamer (HOLMES-Exo_PD-L1_) in the detection procedure. The Aptamer binds to PD-L1, and its glycosylation is less likely to interfere with the antigen than an antibody. It is more sensitive and easier to perform than existing ELISA-based procedures and has a faster reaction time. For the first time, PD-L1 was observed to correlate positively with adenocarcinoma metastasis 83. To increase antibody immobilization, Zhand et al. introduced a protective coating that resulted in an increase in the limit of detection (LOD) and sensitivity that was 225 times and 15.12 times higher than that of commercial ELISA kits, respectively 84. Takeuchi et al. employed PD-1-Ig fusion protein instead of anti-PD-L1 capture antibody in a traditional ELISA to detect 75 plasma samples from patients with NSCLC. The results revealed that it had greater sensitivity and frequency than traditional ELISA 85. Thus, as a standard immunoassay, ELISA plays a significant role in the quantitative detection of PD-L1.

#### 4.1.3. Immunofluorescence

IF uses antigen-antibody specific binding properties and fluorescein labeling techniques to detect PD-L1. The process begins with labeling the antibody with fluorescein, and then binding the labeled antibody to the corresponding antigen. Fluorescence microscopy is then used to determine where the antigen is located. To improve the sensitivity of IF detection, it is crucial to choose substrates that do not release fluorescent chemicals 86. Multiplex immunofluorescence (mIF) staining is currently undergoing development. It permits the detection and nuclear re-staining of as many as six protein markers on a single tissue sample. To differentiate tumor cells from immune cells, Yeong et al. concurrently tagged PD-L1 with three commercially available PD-L1 antibodies (SP142, SP263, and 22C3) and an epithelial cell adhesion molecule (EpCAM). Five-color multiplexed immunofluorescence images of PD-L1 SP142 (green), PD-L1 22C3 (red), PD-L1 SP263 (white), EpCAM (orange), and DAPI (blue) were obtained for tissue sections of triple-negative breast cancer, which effectively detected the PD-L1 content 87. Furthermore, mIF staining has the ability to rapidly and reproducibly identify specific cell populations in the tumor microenvironment; however, few studies have validated this technique against standard clinical immunohistology, which hinders its incorporation into clinical practice. Yaseen et al. compared the ability to detect the expression of immune markers between mIF and conventional chromogenic IHC and single-plex IF. The results showed that mIF accurately detected the expression of immune cell markers (CD8, CD68, and CD16), immune checkpoint PD-L1, and melanoma marker SOX10, with better accuracy and reproducibility than IHC and single-plex IF. This study demonstrates the potential for the use of mIF in clinical practice 88.

Finally, there has been rapid development of artificial intelligence-based imaging analysis techniques in recent years. Vahadane et al. combined artificial intelligence techniques with mIF imaging to design an automated combined positive score (CPS) scoring pipeline to assess PD-L1 expression in tumor biopsies. This study showed that automated CPS scoring using artificial intelligence on trichrome mIF images correlated well (78%, p = 0.003) with manual CPS scoring by pathologists on two-color PD-L1 chromogen IHC images. The clinical application of the technology could greatly reduce the burden on pathologists and make the task of scoring CPS less time-consuming and easier to perform. However, the technique still has some shortcomings and cannot effectively identify some artifacts, such as extrusion artifacts and PD-L1 staining necrosis 89.

### 4.2. Electrochemical detection

Quantitative detection of PD-L1 using an electrochemical immunoassay is a promising method, which has the advantages of accuracy, non-invasiveness, rapidity, and real-time monitoring compared to conventional immunoassays for clinical practice; however, such methods are still scarcely reported due to their novelty. Voltammetry is a commonly used method for electrochemical sensors, including cyclic voltammetry and differential pulse voltammetry. The latter has higher sensitivity and lower detection limits due to reduced background currents 90.

**Figure 3:**
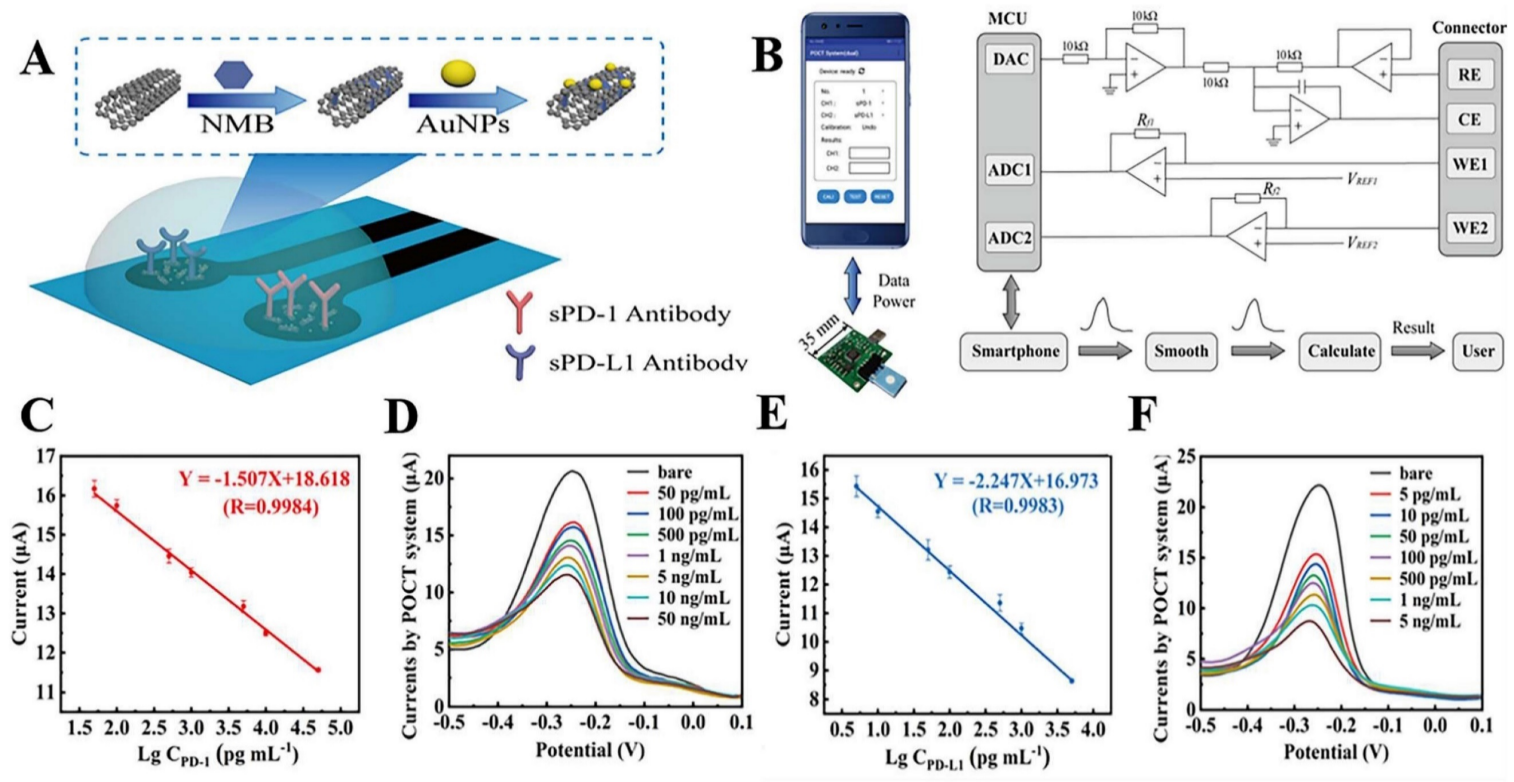
** (A)** Modification of the working electrode. **(B)** Illustration of the POCT system. **(C)** Calibration curve between the peak current and the logarithm of sPD-1 concentrations. **(D)** DPV responses of different sPD-1 concentrations using the POCT system. **(E)** Calibration curve between the peak current and the logarithm of sPD-L1 concentrations. **(F)** DPV responses of different sPD-L1 concentrations using the POCT system. (Cited from reference 94, with permission by ACS.)

The innovative electrochemical immunoassay approach is based on conventional immunoassay methods. The antibodies and nucleic acid Aptamers of conventional immunoassays are the most prevalent biometric molecules in electrochemical immunoassays. An Aptamer is a short oligonucleotide sequence that can be separated from a single-stranded DNA or RNA library. It has a distinct three-dimensional structure that is easily modifiable, cost effective, heat resistant, and durable 91**.** Due to the advantages of Aptamers, electrochemical Aptamer sensors typically exhibit good stability and sensitivity 92,93. Xing et al. employed nucleic acid Aptamers to detect sPD-L1 levels in serum. The research produced nanocomposites composed of amine-functionalized single-walled carbon nanotubes (NH_2_-SWCNT), new methylene blue (NMB), and gold nanoparticles (AuNPs). NH_2_-SWCNTs were physically stacked on the carbon electrode's surface, which enhanced the electrode's conductivity and offered anchoring sites for NMB and AuNPs. NMB was attached to NH_2_-SWCNT via π-π bonds, and AuNPs were directly bonded to the amino groups of NH_2_-SWCNT. To enable electrochemical detection of sPD-L1, nucleic acid Aptamers were linked to AuNPs by modified sulfhydryl groups and specifically captured sPD-L1. This method had an LOD of 10 pg.mL^-1^, a linear detection range of 10 pg.mL^-1^ to 2.5 ng.mL^-1^, and a coefficient of determination of 0.9977 90. In another study, Xing et al. improved the above detection method by fabricating a dual-channel electrochemical detection platform for the rapid and simultaneous detection of sPD-1 and sPD-L1 concentrations in liquids. The platform also uses nanocomposites consisting of NH_2_-SWCNT, NMB, and AuNPs to modify the working electrode. However, they chose PD-L1 and PD-1 antibodies as biometric molecules for the specific detection of sPD-L1 and sPD-1 in liquids **(Fig. 3 A)**. Remarkably, they created a portable electrochemical detection system** (Fig. 3 B)**, referred to as POCT, enabling electrochemical detection with a smartphone. The method uses a small amount of sample, and the results can be obtained on the smartphone in about 20 min. Compared to ELISA, this method offers a short analysis time, portability, and automatic data processing and display of results for sPD-1 and sPD-L1 in serum and plasma. These research findings showed that sPD-L1 had an LOD of 5 pg.mL^-1^ and a linear range of 5 pg.mL^-1^ to 5 ng.mL^-1^, whereas sPD-1 had an LOD of 10 pg.mL^-1^ and a linear range of 50 pg.mL^-1^ to 50 ng.mL^-1^ 94 **(Fig. 3 C-F)**. In another study, Moazzam et al. used PD-L1 antibody (Ab1)-modified gold-coated magnetic nanoparticles (Au@MNPs) as “dispersion electrodes” to achieve rapid, ultrasensitive, and selective electrochemical detection of PD-L1 in undiluted whole blood. Compared to commercial ELISA kits, this method not only greatly reduces the test time but also increases the detection limit by 260,000-fold. Nonetheless, the problem of increased uncertainty persists when working with undiluted whole blood relative to measurements in buffers. A wide linear range of 1.38 aM ~ 13.8 pM was seen in PBS solution, while a range of 13.8 aM ~ 0.138 nM was seen in untreated whole blood 95.

**Figure 4:**
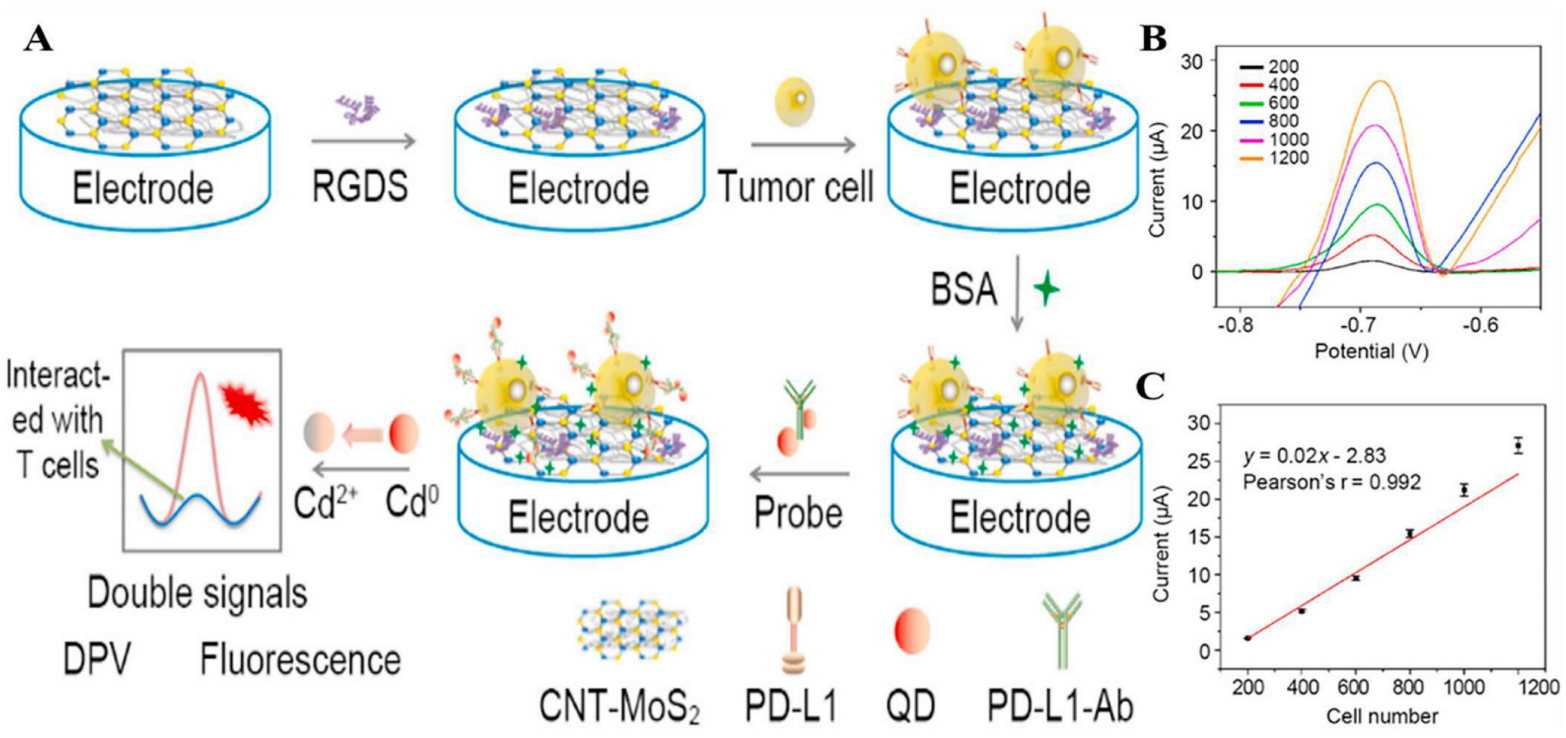
** (A)** Construction of the PD-L1 electrochemical biosensor.** (B)** DPV detection of varying numbers of A375 cells with the PD-L1-QD biosensor.** (C)** Calibration curve showing the linear relationship of the biosensor for the detection of A375 cells. (Cited from reference 97, with permission by Elsevier.)

**Figure 5:**
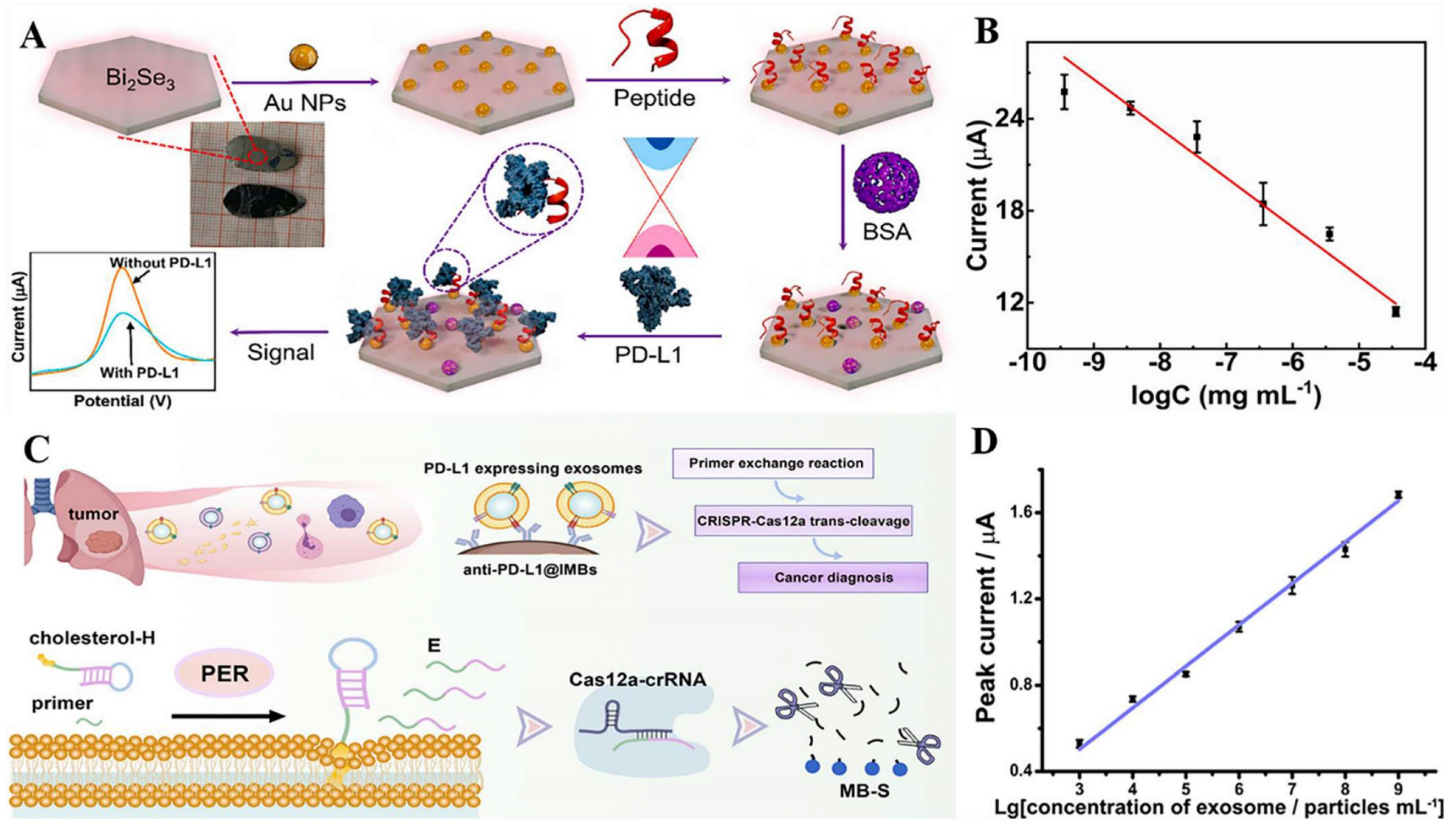
**(A)** Schematic illustration for the detection of PD-L1 using peptide-coated electrochemical biosensor based on Bi_2_Se_3_.** (B)** Calibration plot between the DPV peak current and the logarithm of the PD-L1 concentration. **(C)** Working Principle for Electrochemical Analysis of PD-L1-expressing Exosomes by Using Programmable DNA Fueled Cascade Signal Amplification Reaction. **(D)** Linear relationship between the peak currents and the logarithmic values of exosome concentrations. (A and B cited from reference 99, with permission by Elsevier; C and D cited from reference 103, with permission by ACS.)

The improvement of biosensor performance depends on the selection of an electrode material with good conductivity and stability. MoS_2_ is a graphene-like layered nanostructure with a large surface area, high electrical conductivity, good stability, and easy functionalization, which shows great potential for application in electrochemical sensors 96. Du et al. developed a novel electrochemical sensor that could specifically recognize PD-L1 expressed by living tumor cells and tissues; the sensor was based on the nanomaterial MoS_2_ and used the PD-L1 antibody as a biorecognition molecule** (Fig. 4 A)**. They modified the surface of a glassy carbon electrode with a nanocomposite made of multi-walled carbon nanotubes and MoS_2_, then added RGDS to promote cell adhesion. They also designed a probe that uses both fluorescent and electrochemical signals to detect PD-L1 antibodies. This method can directly detect PD-L1 in tumor tissue without needing to fix or process the sample, which can affect the results 97** (Fig. 4 B and C)**. MoS_2_ can accumulate in a way that blocks signal transmission when modified on the electrode surface. Mao et al. solved this issue by arranging the MoS_2_ vertically to create a B-Pep-MoS_2_┴GO-ITO electrochemical sensor for the rapid detection of the PD-L1 protein in serum**.** They used a hydrothermal method to create MoS_2_-GO nanocomposites with vertical structures on the ITO surface and attached a designed PD-L1-binding peptide (B-Pep) to specifically bind PD-L1. The vertical arrangement of MoS_2_ provides more attachment sites for B-Pep and improves the protein capture ability of the MoS_2_ electrode. In addition, the vertical arrangement of MoS_2_ enhances the electron transfer between the electrode and the electrolyte, which improves the sensitivity of the electrochemical sensor. The LOD of PD-L1 was 2 ng.mL^-1^, and the linear range was 25 ng.mL^-1^ to 500 ng.mL^-1^ 98. Furthermore, Jiang et al. fabricated an electrochemical sensor comprising the topological insulator material Bi_2_Se_3_ and a peptide **(Fig. 5 A)**, which can be used to detect PD-L1 in serum. The AuNPs were first dispersed onto the surface of Bi_2_Se_3_, and then the self-assembled PD-L1-targeting peptide was modified onto the AuNP/Bi_2_Se_3_ electrode surface by cysteine. The peptide/AuNP/Bi_2_Se_3_ electrode was then treated with BSA, resulting in a BSA/peptide/AuNP/Bi_2_Se_3_ working electrode. This study enhanced electron transport between the electrode and electrolyte interface by harnessing the large working area and homogeneous properties of the Bi_2_Se_3_ sheet. Additionally, targeting peptides are easier to synthesize and chemically modify compared to antibodies, which makes them more capable of specifically recognizing and capturing PD-L1. These advantages lead to better specificity, stability, and selectivity of the sensor. The LOD of the method was 1.07 × 10^-11^ mg.mL^-1^, and the linear detection range was 3.6 × 10^-10^ mg.mL^-1^ to 3.6 × 10^-5^ mg.mL^-1^ 99** (Fig. 5 B)**.

PD-L1 exists not only on the cell surface but also on the surface of exosomes, and an increasing number of studies have shown that exosomal PD-L1 can suppress immune responses and promote tumor progression 100,101,102. However, electrochemical immunoassay methods for exosome PD-L1 detection are relatively rare. A study by Sha et al. established a programmable DNA-fueled electrochemical assay that can be used to determine the number of PD-L1-expressing exosomes in lung cancer **(Fig. 5 C)**. First, PD-L1-expressing exosomes were bound to PD-L1 antibody-functionalized immunomagnetic beads (anti-PD-L1@IMBs), and a cholesterol-modified hairpin template then interacted with the exosomes. The catalytic hairpin then underwent a primer exchange reaction (PER), which generated a large number of extended primers that activated the trans-cleavage activity of the Cas12a protein. The activated Cas12a protein catalyzed the degradation of the methylene blue-labeled signal strand, generating several methylene blue-labeled short DNA fragments. Finally, MB molecules were captured by a cucurbit[7]uril-modified electrode, producing a distinct electrochemical signal. The method achieved cascade amplification of the signal through the trans-cleavage reaction promoted by PER and Cas12a, which improved the detection sensitivity. The detection range of exosomes was from 10^3^ to 10^9^ particles.mL^-1^, with a low detection limit of 708 particles.mL^-1^ 103 **(Fig. 5 D)**.

### 4.3. Photochemical detection

#### 4.3.1. Surface plasmon resonance immunoassay

Surface plasmon resonance (SPR) technology is an emerging technology in the field of fiber optic sensing. The technique involves coating the surface of the fiber's core with a thin layer of metal, and then exciting surface plasmon waves on the metal surface when light is coupled to the fiber 104. Due to its large specific surface area, excellent optoelectronic properties, highly ordered structure, good dispersibility, excellent biocompatibility, short response time, and real-time detection 105, the SPR technique is widely used for the rapid and sensitive detection of trace disease markers. In recent years, the SPR approach for the immunoassay of PD-L1 content has emerged and demonstrated success.

**Figure 6:**
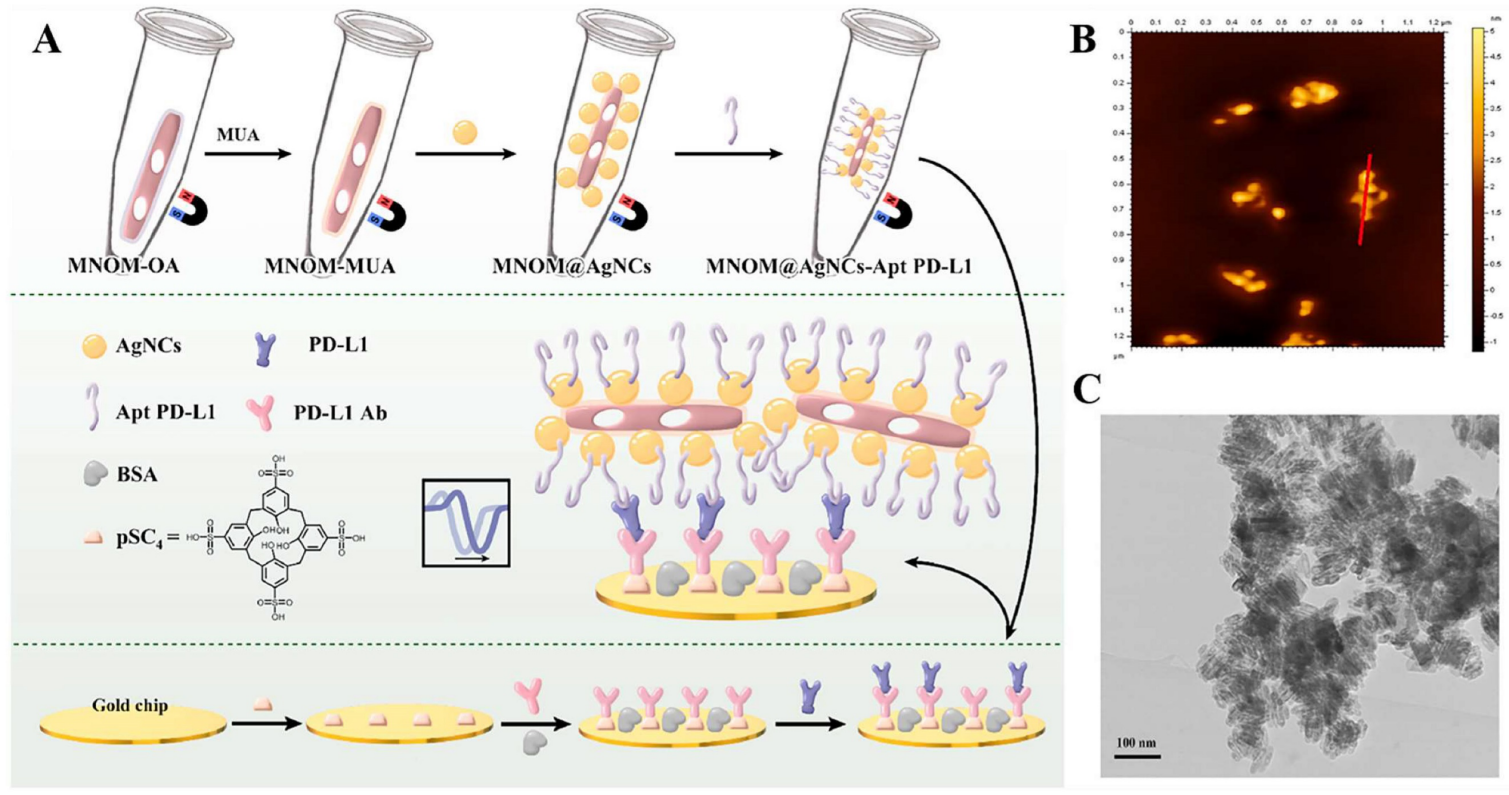
** (A)** Schematic diagram of MNOM@AgNCs-Apt PD-L1 magneto-optical nanocomplex with enhanced sensitivity to detect PD-L1.** (B)** 2D AFM images e of MNOM@AgNCs. **(C)** TEM image of MNOM@AgNCs. (Cited from reference 107, with permission by Elsevier.)

**Figure 7:**
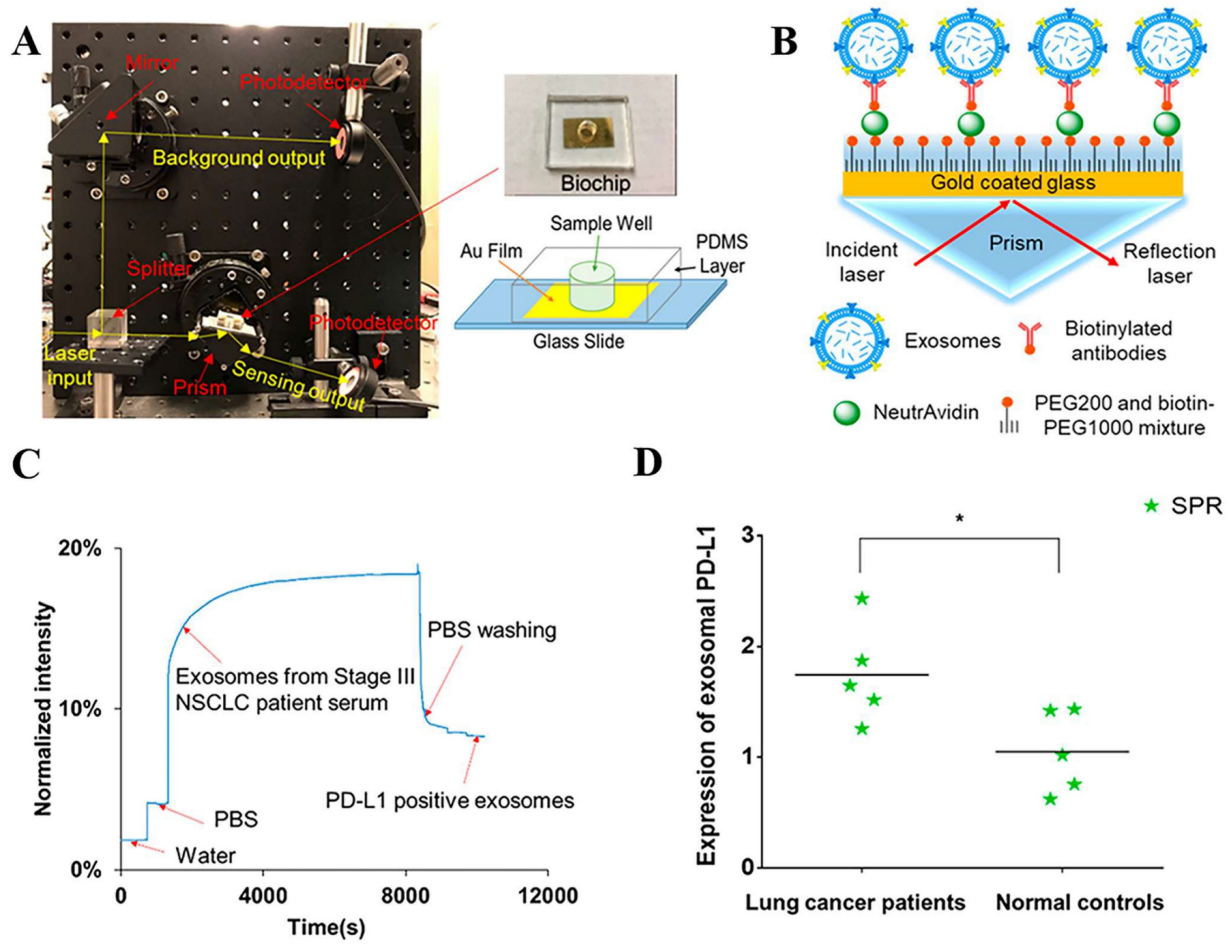
** (A)** Setup of compact SPR biosensor (left) and the photo and schematic diagram of the biochip (right).** (B)** Sensing mechanism of compact SPR biosensor. **(C)** Representative real-time response curve of compact SPR biosensor detecting exosomal PD-L1 in serum sample from a Stage III lung cancer patient.** (D)** Expression of exosomal PD-L1 in serum samples measured by compact SPR biosensor. ELISA was not able to detect exosomal PD-L1 levels in serum samples. (Cited from reference 111, with permission by ACS.)

**Figure 8:**
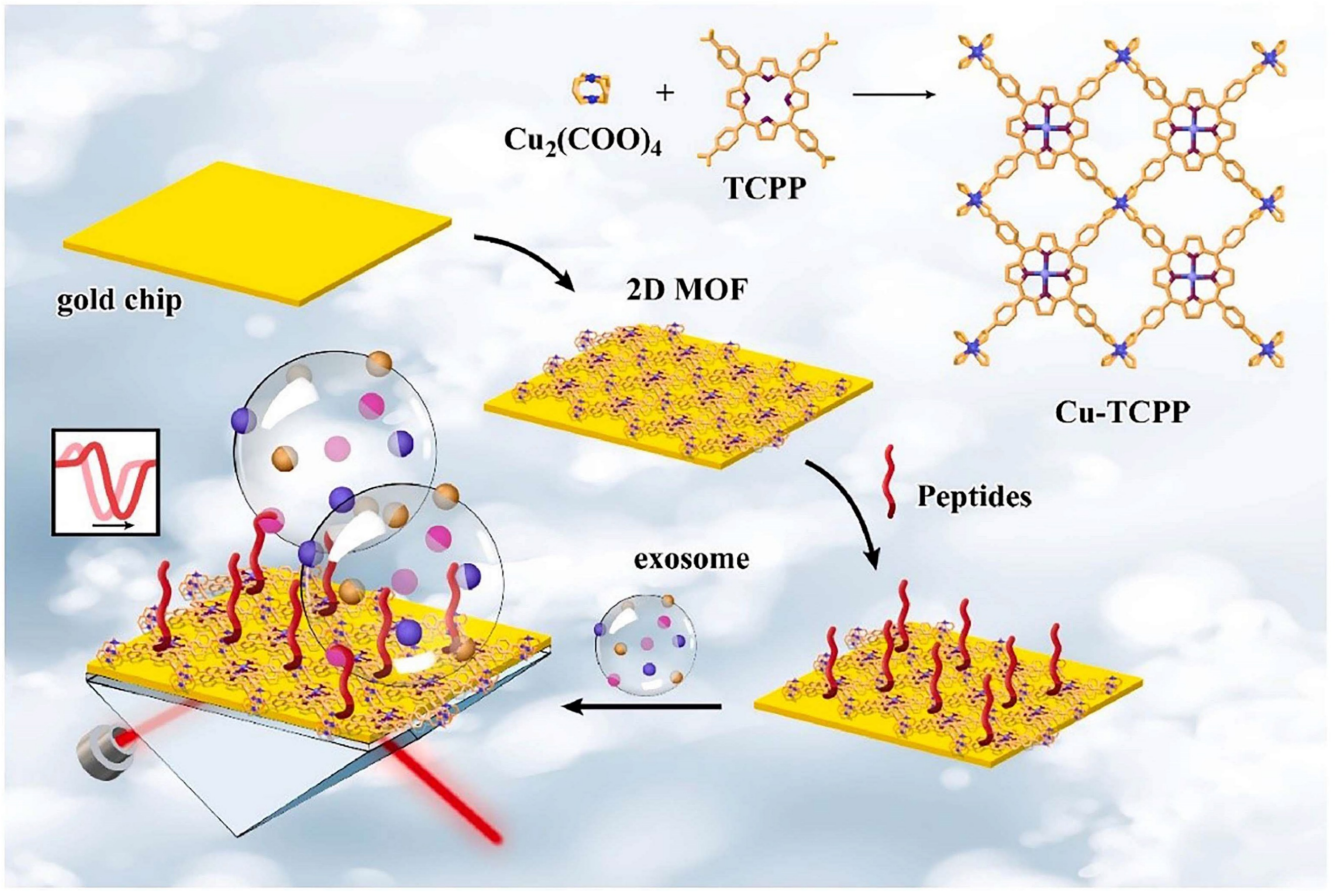
Schematic diagram of 2D MOF-based SPR biosensor for simple and ultra-sensitive detection of PD-L1 exosomes. (Cited from reference 113, with permission by Elsevier.)

The PD-L1 protein is expressed on the cell membrane, on the surface of exosomes, in the nucleus, and as a soluble circulating protein (sPD-L1) 106. Currently, SPR-based PD-L1 assays enable the simultaneous detection of a specific type or multiple types of PD-L1. Hang et al. created an easy and highly specific SPR biosensor for the detection of PD-L1 in human plasma based on magnetite nanorods containing ordered mesocages (MNOM) and silver nanoclusters (AgNCs)** (Fig. 6 B and C)**. However, the type of PD-L1 protein was not specified in this study. The PD-L1 antibody on the gold chip and the PD-L1 Aptamer on the MNOM@AgNC could achieve dual-selective recognition of PD-L1 to increase the sensor's selectivity and decrease nonspecific binding **(Fig. 6 A)**. This technique had a linear range of 10 ng.mL^-1^ to 300 ng.mL^-1^ and a detection limit of 3.29 ng.mL^-1^ for PD-L1 detection, which was verified in healthy and cancer patient test samples, providing a pathway for clinical real-time PD-L1 detection 107. sPD-L1 is not anchored in the plasma membrane or vesicles, but is free in solution; thus, it can be detected in the serum of patients with cancer, autoimmune diseases, or certain viral diseases, as well as other conditions. However, its detection is limited due to its low concentration in serum and various body fluids 108. Hu et al. constructed a simple and sensitive SPR sandwich assay to detect serum sPD-L1 based on the unique and strong binding ability of a specific intracellular binding peptide (SIBP) to the intracellular region of sPD-L1. In this study, pSC4 was immobilized on a gold chip, and then the anti-PD-L1 antibody was specifically bound to pSC4. By binding to anti-PD-L1, sPD-L1 could be immobilized on the surface of gold electrodes. Given the natural binding affinity of SIBP to the intracellular region of sPD-L1, further signal amplification with AuNPs-SIBP (100 μg.mL^-1^) was successfully achieved for the detection of sPD-L1, and the dynamic response range of sPD-L1 concentration reached 10-2000 ng.mL^-1^ 109. Exosomal PD-L1 is another important form of PD-L1 present extracellularly and may be a more reliable marker than tumor biopsy PD-L1 expression 110. Currently, most SPR-based PD-L1 assays are for exosomal PD-L1. One study described a new type of SPR biosensor (25 × 10 × 25 cm) that is both small in size and intensity-modulated, making it more practical for clinical applications than other commercial SPR sensors that are larger, more expensive, and less clinically applicable. The biosensor uses a conventional SPR sensing mechanism but does not require nanostructure fabrication to achieve exosomal PD-L1 detection **(Fig. 7 A and B)**. It also has a sensitivity of 9.258*10^3^% RIU and a resolution of 8311*10^-6^ RIU. Using this biosensor, exosomal PD-L1 expression was detected considerably better in patients with NSCLC than in normal controls** (Fig. 7 C)**, and the detection sensitivity was greater than that of ELISA 111** (Fig. 7 D)**. Zhang et al. immobilized a biotinylated PD-L1 Aptamer on a streptavidin sensor chip, achieving label-free detection of exosomal PD-L1 based on surface plasmon resonance (SPR-ExoPD-L1). In addition, they used iodixanol density gradient centrifugation for the isolation and purification of exosomes before the assay. This PD-L1 assay has the advantage of using iodixanol density gradient centrifugation to exclude major non-vesicular components and large extracellular vesicles, and the Aptamer can more easily penetrate the glycosylated PD-L1 protein of exosomes, which increases the sensitivity of detection. The method can distinguish not only tumor exosomes from normal exosomes but also exosomes with different PD-L1 expression levels 112. Cu-TCPP 2D MOF was added to the experiment by Wang et al. to increase the sensitivity of exosomal PD-L1 detection (**Fig. 8**). With 2D MOF-modified gold chips, the refractive index sensitivity, detection precision, and quality factor all showed considerable improvements. The LOD of the SPR sensor was 16.7 particles.mL^-1^. Analysis of exosomal PD-L1 in human serum samples served to further establish its validity and applicability 113.

#### 4.3.2. Localized surface plasmon resonance immunoassay

In the past few decades, localized surface plasmon resonance (LSPR) sensors have attracted significant attention in the biological, chemical, and environmental monitoring fields because of their high sensitivity to the surrounding refractive index 114,115. In contrast to SPR on a single substrate (e.g., a thin metal film), the plasma of LSPR is generated on metal nanoparticles 116. The LSPR effect can be induced by exciting the surface of metal nanoscale particles with incoming light. It produces a locally increased light field, and the plasma is confined to nearby nanostructures. Therefore, LSPR technology is more localized and permits detection processes at the interface of the platform. Simultaneously, it has greater spatial sensitivity than standard SPR biosensors 117. LSPR for detecting PD-L1 content has comparatively few reports compared to SPR; however, it offers promising research opportunities due to its advantages.

**Figure 9:**
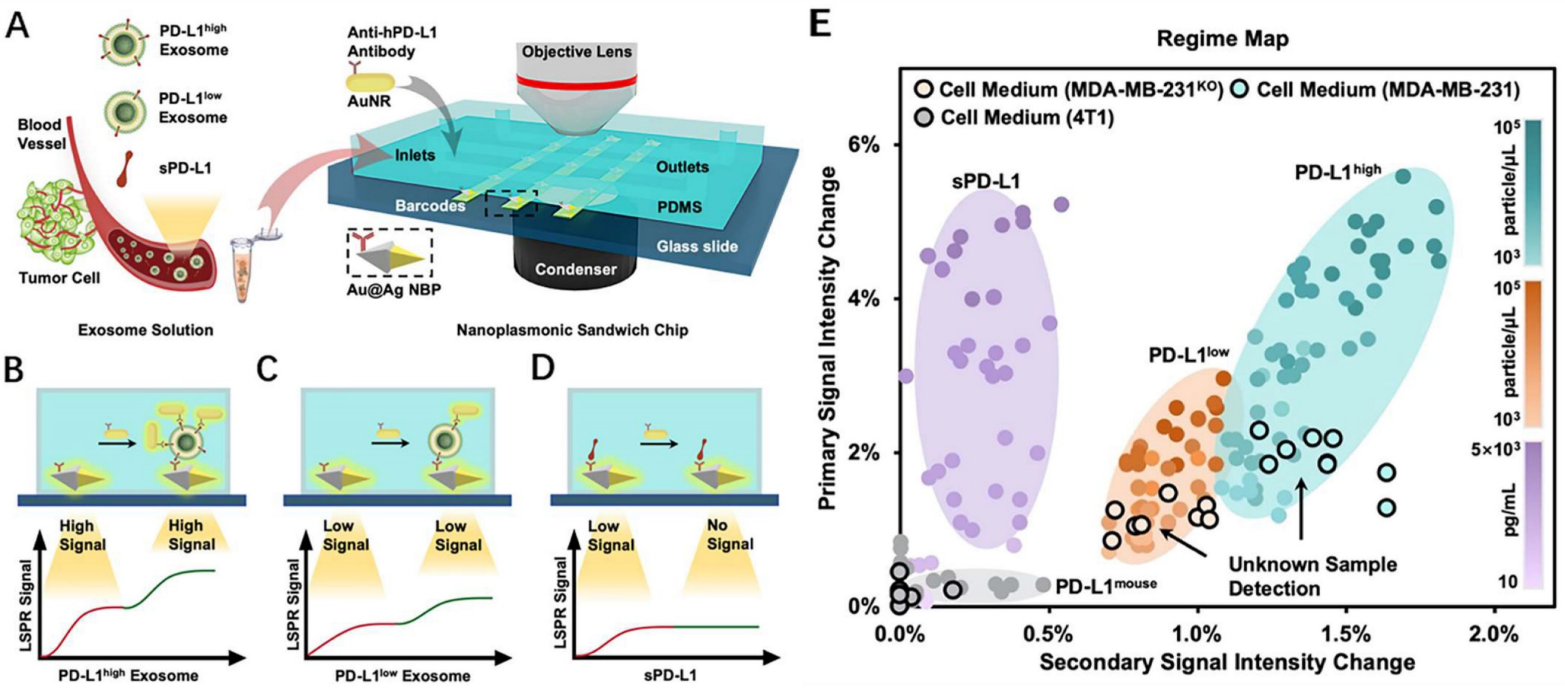
** (A)** Detection procedure of the nanoplasmonic sandwich immunoassay.** (B-D)** Quantification and subclass identification of exosomes based on the generated primary (red curves) and secondary (green curves) LSPR signals. **(E)** Regime map for exosome subtyping and unknown sample detection. (Cited from reference 119, with permission by ACS.)

During transverse LSPR (t-LSPR) and longitudinal LSPR (l-LSPR), gold nanorods (AuNRs) form two absorption bands. The l-LSPR band is currently the dominant absorption band, and its position can be changed by modulating the aspect ratio of AuNRs 118. Using AuNR, Wang et al. quantified exosomal sPD-L1 content. This method functionalized the gold-silver core-shell nanomaterials with anti-PD-L1 antibodies, which was the main trapping layer in the sandwich structure. Binding with exosomal sPD-L1 in the loaded sample increases the redshift and scattering intensity of the plasmon resonance wave, resulting in a primary LSPR signal. In addition, AuNRs demonstrated robust scattering during dark-field imaging, resulting in a secondary LSPR signal **(Fig. 9 A-D)**. In contrast to existing assays, this technique not only achieves a highly sensitive assessment of exosomal sPD-L1 content but also permits subtype typing of exosomes using different optical correspondences of primary and secondary signals. It can differentiate exosomes with varying levels of PD-L1 expression—PD-L1^low^, PD-L1^high^, and PD-L1^mousen^—and has been verified in MDA-MN-231 human breast cancer cells by comparison with flow cytometry typing 119 **(Fig. 9 E)**. For LSPR immunoassays of PD-L1 content, large gold nanoshells (160 nm in diameter) were also utilized. Luo et al. built an ExTFG-LSPR biosensing platform. This study utilized anti-sPD-L1 mAb to functionalize the sensor for the label-free, fast, and selective detection of sPD-L1. The immunosensor was highly selective for sPD-L1 and could recognize sPD-L1 at low concentrations, with an LOD of around 1 pg.mL^-1^ (0.04 pM) for the target sPD-L1 molecule. In addition, the sensitivity of the sensor could be enhanced by lowering the core/cladding diameter of the ExTFG to fulfill the significantly lower LOD requirement 120.

### 4.4. Nuclear medicine imaging

**Figure 10:**
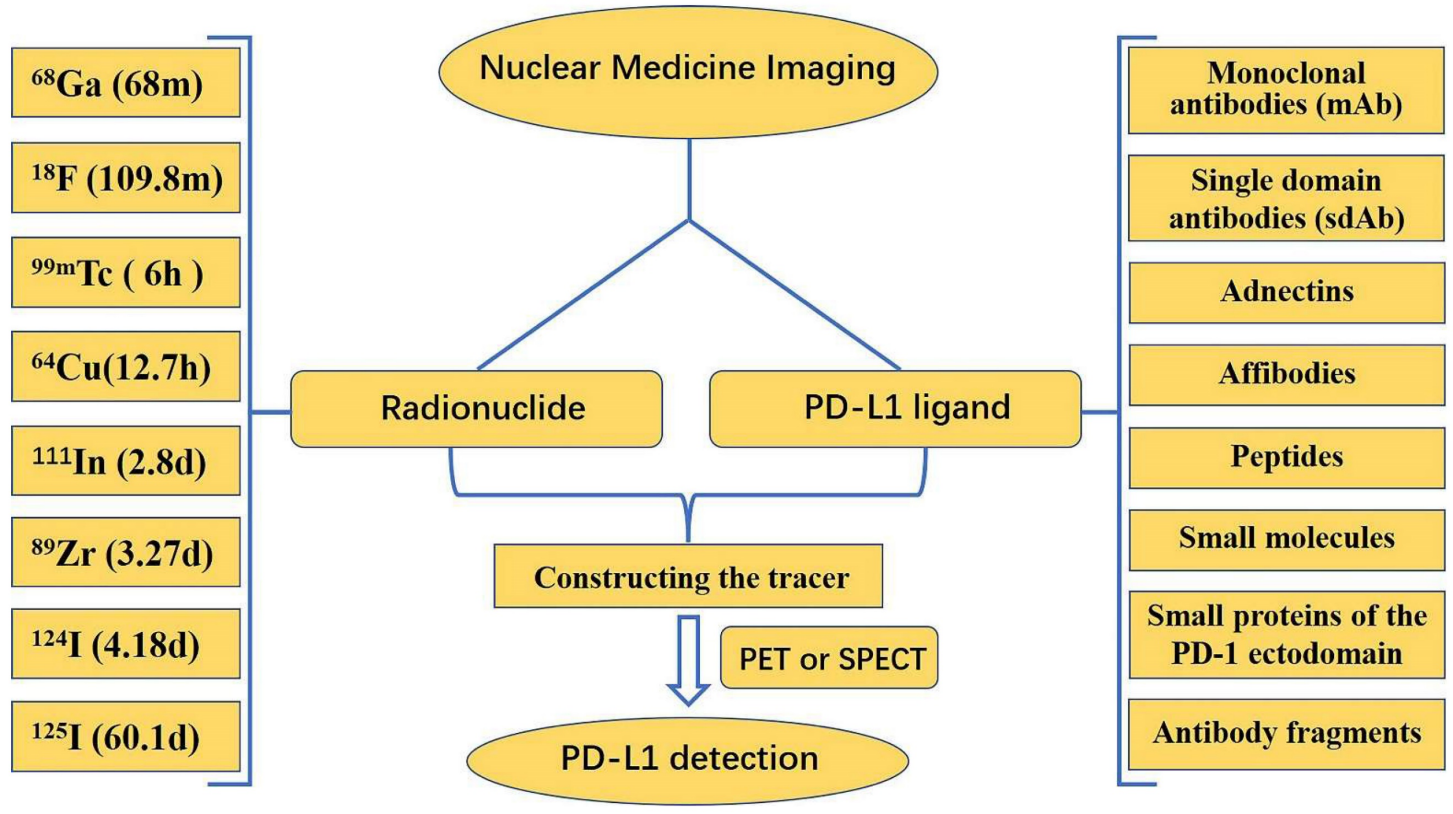
This framework describes the main radionuclides and PD-L1 ligands for the detection of PD-L1 in PET/SPECT imaging.

The expression of PD-L1 molecules in tumors is very dynamic and has some spatial and temporal heterogeneity. Standard methods, such as IHC and ELISA, are often insufficient to accurately and comprehensively assess PD-L1 expression. Nuclear medicine imaging appears to be a promising approach to address this problem by achieving accurate, comprehensive, and dynamic detection of PD-L1 expression levels in tumors in a non-invasive manner. Both primary tumors and metastases can be evaluated by a uniform noninvasive whole-body imaging procedure, thus avoiding sampling errors in the presence of PD-L1 expression heterogeneity. In addition, nuclear medicine imaging allows for a more accurate quantitative measurement of total PD-L1 expression in individual patients, which can be used to determine appropriate therapeutic approaches and dosages for anti-PD-L1 therapies 121. Currently, nuclear medicine imaging relies mostly on positron emission tomography (PET) and single photon emission computed tomography (SPECT). In the detection of PD-L1 using nuclear medicine imaging, research is mainly focused on the development of new tracers, which are composed of two parts: a radionuclide, and a PD-L1 ligand **(Fig. 10)**.

Numerous radionuclides have been utilized in the labeling of ligands for the production of various tracers. Each radionuclide has a different half-life: ^68^Ga has a half-life of 68 min,^ 18^F has a half-life of 109.8 min,^ 99m^Tc has a half-life of 6 h,^ 64^Cu has a half-life of 12.7 h,^ 111^In has a half-life of 2.8 d, ^89^Zr has a half-life of 3.27 d,^ 124^I has a half-life of 4.18 d, and ^125^I has a half-life of 60.1 d. Long half-life radionuclides such as ^111^In and ^89^Zr, which have similar half-lives to the biological half-lives of mAbs, are beneficial for imaging; however, long half-life radionuclides have drawbacks, such as delayed clearance, long imaging times, and excessive radiation doses to healthy organs 122. Consequently, it is essential to choose the proper radionuclide when manufacturing tracers. Among PET-based and SPECT-based tracer constructs, ^18^F is commonly utilized in PET, whereas ^99m^Tc is used more frequently in SPECT.

#### 4.4.1. Monoclonal antibodies

mAbs have proven to be highly reliable PD-L1 ligands because of their high specificity, high affinity, and availability. In 2015, Natarajan et al. reported the first study using PET imaging of PD-1 expression in living subjects with tracers. They developed a tracer based on an anti-mouse PD-1 mAb, ^64^Cu-DOTA-PD-1, to detect PD-1 expression in a transgenic mouse model of melanoma. They discovered that mice receiving the anti-PD-1 tracer exhibited high radiolucency signals in lymphoid organs and tumors. However, when binding was blocked by unlabeled antibodies, the radiation signal was reduced by a factor of two. The strategy achieved the first successful detection of PD-1 by PET imaging *in vivo* 123. In the same year, Heskamp et al. for the first time, showed PD-L1 expression in tumors using SPECT/CT imaging. They injected PD-L1.3.1, an anti-PD-L1 antibody labeled with Indium-111 (^111^In), into a xenograft mouse model, and then used SPECT/CT imaging to analyze PD-L1 expression in the mouse tumors. They discovered that non-invasive detection of PD-L1 in cancer lesions is possible in mice using SPECT/CT imaging with ^111^In-PD-L1.3.1 124. In 2018, Bensch et al. conducted a first-in-human study designed to assess the feasibility of PET imaging with zirconium-89-labeled atezolizumab (anti-PD-L1) and examined the potential of this approach to predict the PD-L1 blockade response. Preliminary results suggested that assessment of PD-L1 status using molecular PET imaging could better predict clinical response in patients compared to predictive biomarkers of immunohistochemistry or RNA sequences. This finding encouraged further research and development of molecular PET imaging techniques to better assess PD-L1 status and treatment response in oncology patients 125. Later, several novel ^89^Zr-labeled tracers emerged, including [^89^Zr]Zr-DFO-PD-L1 mAb 126, ^89^Zr-DFO-6E11 127, and [^89^Zr]DFO-Anti-PDL1^128^, demonstrating that ^89^Zr-labeled mAb-based tracers have some clinical translation potential. In addition, Li et al. reported a humanized full-length anti-PD-L1 mAb probe labeled with ^124/125^I: ^124/125^I-CS1001, and their results demonstrated the feasibility of its use for monitoring and assessing PD-L1 expression in tumors 129. As demonstrated above, mAbs have distinct benefits in the construction of radiotracers. Nevertheless, due to drawbacks, such as their large molecular size and extended half-life, as well as the slow production of high-contrast pictures, an increasing number of studies are looking for tracers with low molecular weight, rapid clearance, and high tumor uptake. The non-mAb PD-L1 ligands currently under investigation are monodomain antibodies (sdAb), Adnectins, Affibodies, peptides, small molecules, small proteins, and antibody fragments.

#### 4.4.2. Single-domain antibodies

**Figure 11:**
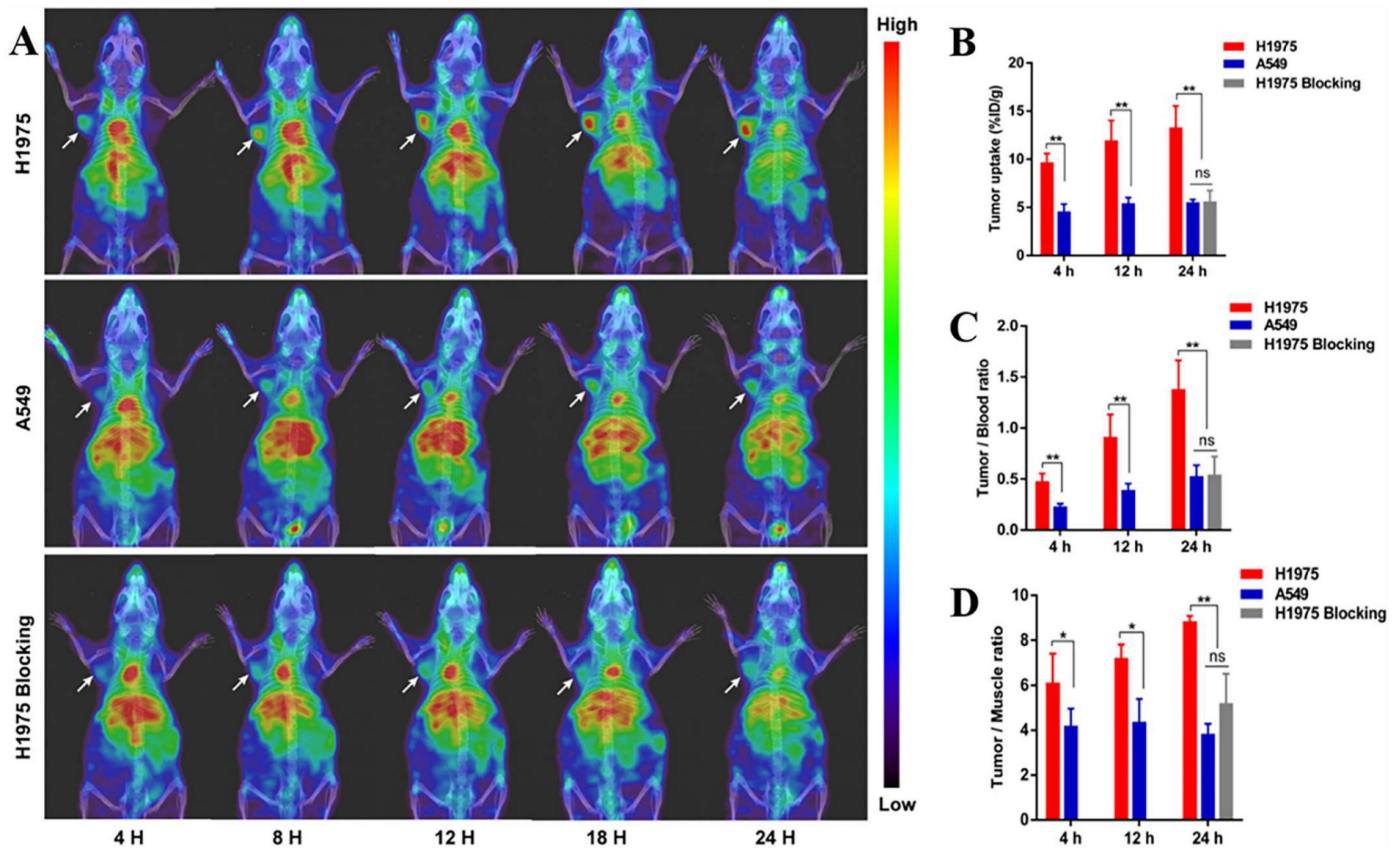
** (A)** SPECT/CT images of H1975, A549, and H1975 blocking tumor-bearing mice at 4, 8, 12, 18, and 24 h administered with [^99m^Tc]Tc-HYNIC-KN035.** (B)** Tumor uptake of [^99m^Tc]Tc-HYNIC-KN035 in H1975, A549, and H1975 blocking groups. The tumor/blood ratio** (C)** and tumor/muscle ratio** (D)** in H1975, A549, and H1975 blocking groups. (Cited from reference 133, with permission by ACS.)

Single-domain antibodies are a promising class of imaging agents because of their small size, ease of engineering, and high binding power to target proteins 130. Broos et al. developed a small (15 kDa) single-domain antibody with high specificity and affinity for PD-L1, named sdAb K2. They found that injecting ^99m^Tc-labeled sdAb K2 into mice produced SPECT/CT images with a high signal-to-noise ratio within 1 h at the earliest. Notably, they also found that sdAb K2 could antagonize the interaction between PD-1 and PD-L1, showing potential therapeutic value 131. In another study, Xing et al. conducted the first human study with a ^99m^Tc-labeled anti-PD-L1 single-domain antibody (NM-01), and they indicated that SPECT/CT imaging was safe using this probe 132. In recent years, Zhang et al. designed an immune SPECT probe named [^99m^Tc]Tc-HYNIC-KN035 for the assessment of PD-L1 expression. The probe was made by conjugating a nanoantibody, KN035, which has high specificity and affinity for PD-L1, to the chelator succinyl 6-hydrazinonicotinate, and then labeling it with the radionuclide ^99m^Tc. Their results showed that [^99m^Tc]Tc-HYNIC-KN035 had a high binding affinity for PD-L1 (K_D_ = 31.04 nM), and its uptake by H1975 cells (high PD-L1 expression) was higher than that of A549 cells (low PD-L1 expression) at all time points. SPECT/CT imaging results showed that [^99m^Tc]Tc HYNIC-KN035 accumulated significantly more in H1975 tumors than in control A549 tumors **(Fig. 11 A)**. Furthermore, [^99m^Tc]Tc-HYNIC-KN035 exhibited consistently high tumor uptake and a satisfactory target-to-background ratio compared with the ^99m^Tc-NM-01 tracer reported by Xing et al. 132. From these results, [^99m^Tc]Tc-HYNIC-KN035 is expected to achieve clinical translation in the future 133** (Fig. 11 B-D)**. Another single-domain antibody, Nb109 (14 kDa), also has a high specific affinity for PD-L1. Chen et al. used a site-specific conjugation strategy to prepare a tracer based on Nb109: ^68^Ga-NODA-CDV-Nb109. Compared to their previously reported ^68^Ga-NOTA-Nb109 121,134, ^68^Ga-NODA-CDV-Nb109 is a homogeneous structure that is more suitable for use in clinical settings. Notably, their results showed that ^68^Ga-NODA-CDV-Nb109 PET imaging could sensitively monitor the upregulation of PD-L1 expression induced by chemotherapeutic agents (Doxorubicin), which facilitated timely interventional therapy. It is important to consider that Doxorubicin treatment may induce systemic toxicity, so it is expected that a less toxic therapy for non-target tissues will be selected in the future to further evaluate whether ^68^Ga-NODA-CDV-Nb109 is suitable for the dynamic monitoring of changes in PD-L1 expression levels in cancer 135.

#### 4.4.3. Adnectins

**Figure 12:**
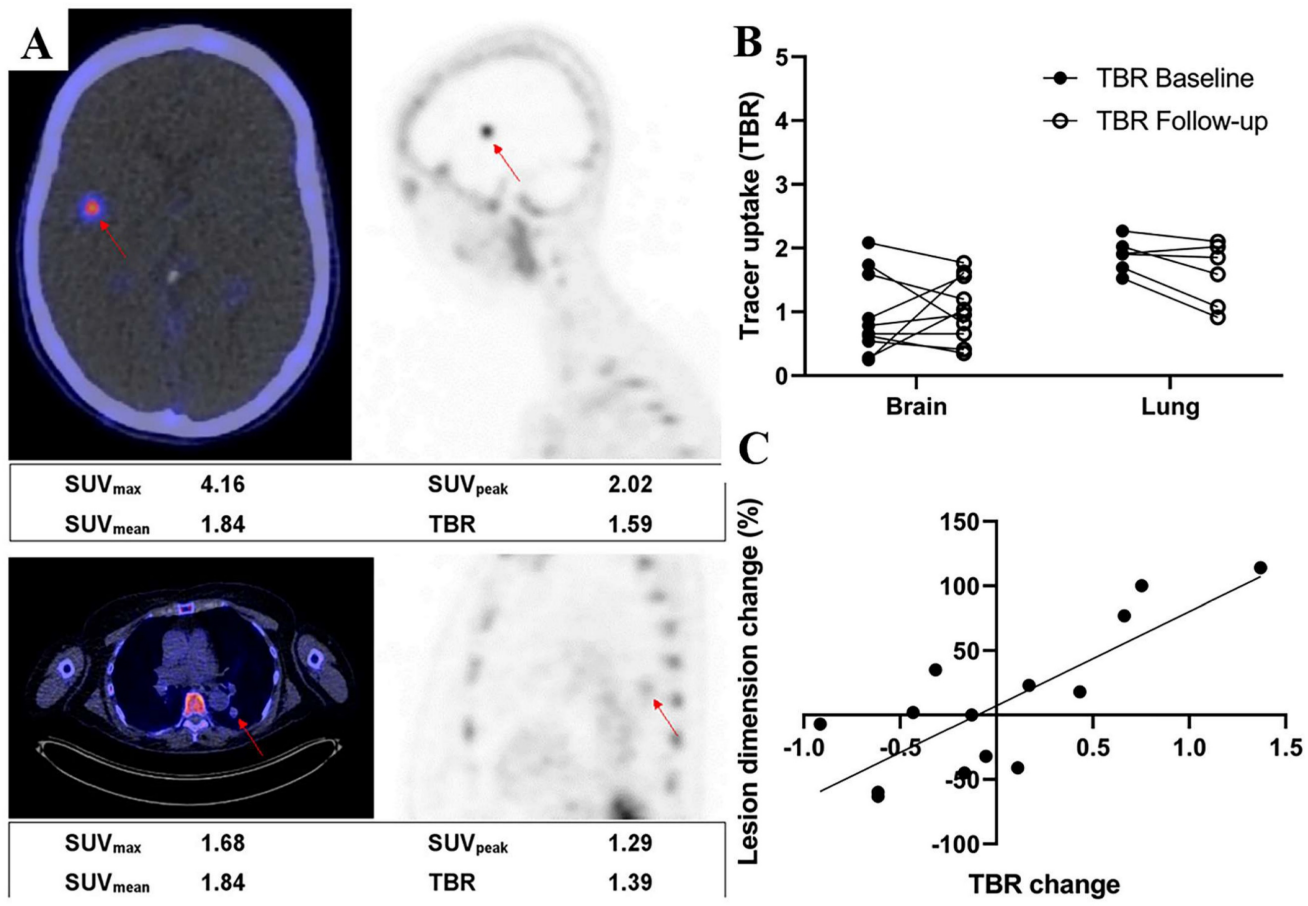
**(A)** Examples of a brain lesion (top) and a lung lesion (bottom). Fused PET/CT transverse images (left) and PET sagittal images (right) are shown. Semiquantitative measurements are reported underneath images. **(B)** ^18^F-BMS986192 uptake for brain and lung lesions at baseline and follow-up (4 patients, 16 lesions). Tracer uptake is reported as TBR. **(C)** Association between change in lesion size and change in TBR of follow-up ^18^F-BMS986192 scan compared with baseline (4 patients, 14 lesions). (Cited from reference 138, this research was originally published in JNM.)

Adnectins are a family of proteins based on ^10^Fn3—the 10th human fibronectin type III structural domain. This domain has the same high binding affinity and specificity as intact antibodies, and it is small in size (~10 kDa) and easily genetically manipulated 136. Donnelly et al. developed a tracer using an Adnectin molecule (BMS-986192) with a high binding affinity for PD-L1, ^18^F-BMS- 986192, which was deemed feasible for the detection of PD-L1 expression in animal tumor models using^ 18^F-BMS-986192 PET 137. Later, Nienhuis et al. explored the relationship between lesion uptake of ^18^F-BMS986192 and tumor response by using ^18^F-BMS-986192 PET to detect PD-L1 expression in patients with metastatic melanoma **(Fig. 12 A)**. In this study, four patients underwent whole-body ^18^F-BMS-986192 PET/CT scans before starting immune checkpoint inhibition treatment. Six weeks after treatment, it was found that the increased uptake of ^18^F-BMS-986192 was significantly associated with increased tumor size at response assessment compared to baseline scans** (Fig. 12 B and C)**. In addition, they found that ^18^F-BMS-986192 PET could detect treatment-induced toxicity at an early stage before the appearance of clinical symptoms. Based on their findings, ^18^F-BMS-986192 PET imaging has great potential clinical value and deserves further investigation 138. However, the process of labeling BMS-986192 with ^18^F is complicated; thus, Robu et al. used ^68^Ga to label BMS-986192 and showed that ^68^Ga-BMS-986192 exhibited comparable PD-L1 targeting specificity and rapid blood clearance to ^18^F-BMS-986192. In comparison to ^18^F-BMS-986192, the synthesis of ^68^Ga-BMS-986192 is significantly simpler and more easily automated 139. In addition, Zhou et al. designed an optimized Adnectin-based tracer against hPD-L1 using 1,4,7-triazacyclononane, 1-glutaric acid-4,7-acetic acid (NODAGA) as a radionuclide chelator (^68^Ga-NODAGA-BMS986192). In comparison to ^68^Ga-NODA-BMS986192 139, they found that the ^68^Ga-NODAGA-BMS986192 tracer with the improved labeling method had higher stability 140.

#### 4.4.4. Affibodies

Affibodies are a class of engineered affinity proteins with a small size (6.5 kDa), high affinity, and high specificity that can be obtained by phage display techniques; they can also be labeled with a variety of radionuclides. From these properties, it is clear that Affibodies have great potential for molecular imaging 141. González Trotter et al. developed an Affibody-based tracer, [^18^F]AlF-NOTA-Z_PD-L1_1_, which was imaged by PET to detect PD-L1 expression within the tumor. Z_PD-L1_1_ in tracers refers to Affibody molecules that bind to PD-L1. They achieved the binding of the [^18^F] radionuclide to Affibodies by maleimide coupling of NOTA with a unique cysteine residue and chelation of ^18^F-AlF. They injected the tracer into immunodeficient mice, and after 90 min, the *in vitro* biodistribution measurements showed that [^18^F]AlF-NOTA-Z_PD-L1_1_ uptake was much higher in LOX (malignant melanoma cell line, PD-L1^+^) tumors than in SUDHL6 (lymphoma cell line, PD-L1^-^) tumors. This suggests that the Affibody molecule Z_PD-L1_1_ has high targeting specificity for PD-L1 expressed in xenograft tumors. Furthermore, they found that the abnormally fast clearance of [^18^F]AlF-NOTA-Z_PD-L1_1_ from the blood may be responsible for the low uptake in the target tissue 142. Their team later improved the Affibody by replacing NOTA-Z_PD-L1_1_ (K_D_ = 1 nM) with the higher affinity NOTA-Z_PD-L1_4_ (K_D_ = 0.07 nM) to create two new tracers, [^18^F]AlF-NOTA-Z_PD-L1_4_ and [^68^Ga]NOTA-Z_PD-L1_4_. They evaluated both tracers in mouse tumor models with PET, and the *in vitro* biodistribution measurements showed that both tracers accumulated 925-fold higher in LOX (PD-L1^+^) tumors than in SUDHL6 (PD-L1^-^) tumors; the lower affinity tracer [^18^F]AlF-NOTA-Z_PD-L1_1_ only showed 8-fold higher accumulation. Notably, PD-L1-expressing lymph nodes in Rhesus monkeys were visible in the PET images of both tracers, reflecting good PD-L1 targeting 143. In addition, Liang et al. prepared a new ^99m^Tc-labeled PD-L1 Affibody molecular probe (^99m^Tc-PDA), which was imaged by SPECT to assess the expression of PD-L1 in tumors. Their results showed that^ 99m^Tc-PDA had a high affinity for MC38-B7H1 cells, with a K_D_ value of about 10.02 nM. Based on their experimental results, they proposed an optimal imaging time of 1-2 h after injection 144. Thus, Affibody-based probes have good affinity and targeting capabilities; however, their uptake is high in the kidney, which limits the uptake at the tumor site and reduces sensitivity in low-expression lesions. Therefore, Affibody probes need to be further investigated to optimize their structure and reduce their uptake in the kidneys.

#### 4.4.5. Peptides and small molecules

**Figure 13:**
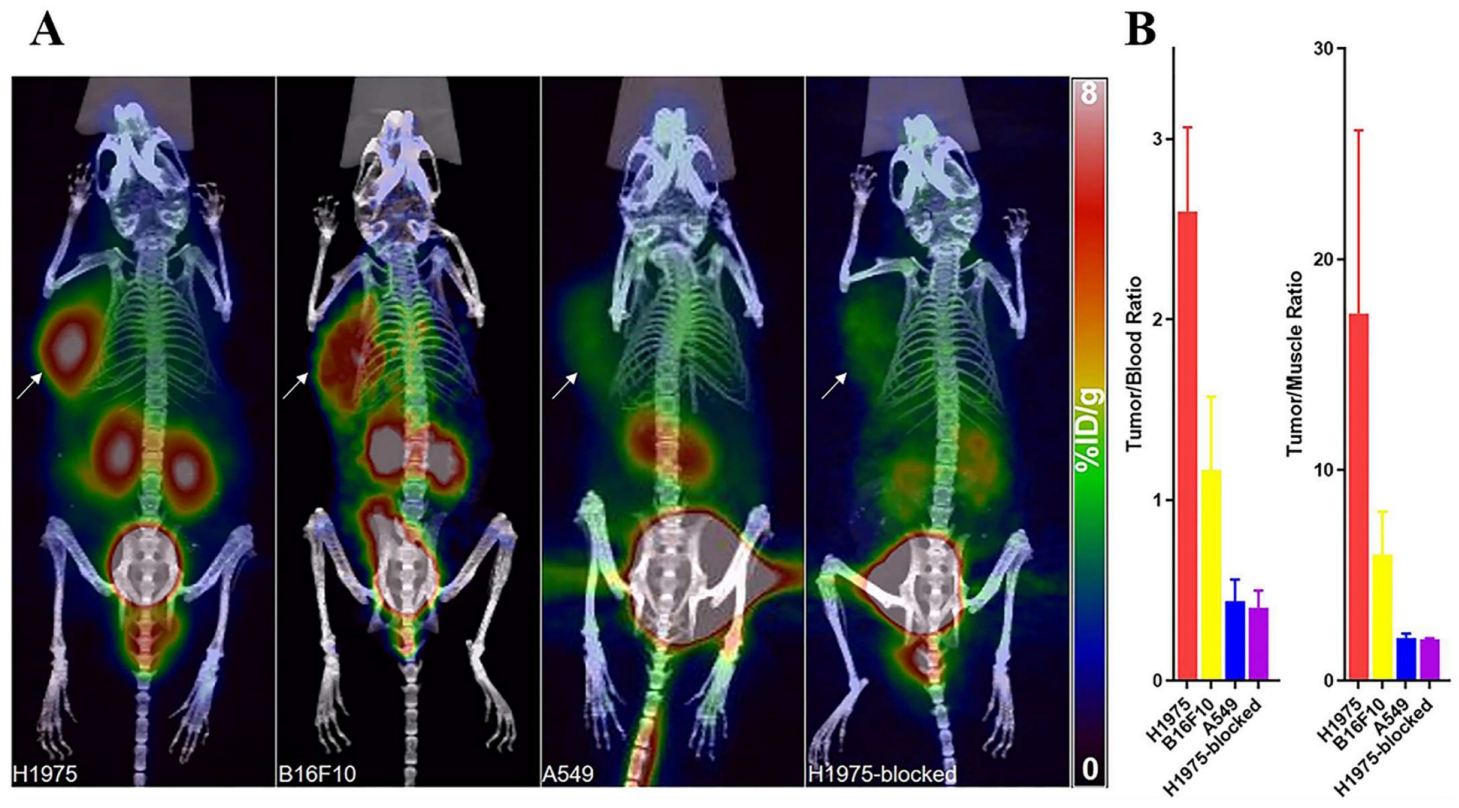
** (A)** PET/CT images of mice with H1975, B16F10, A549 tumors, and mice harboring H1975 tumor with blocking dose at 1 h after the injection. The tumor was indicated by a solid arrow. **(B)** T/B and T/M ratios of H1975, B16F10, and A549 and blocked-H1975 tumor models (n = 3). (Cited from reference 149, with permission by ACS.)

WL12 is a cyclic peptide consisting of 14 amino acids that have a high binding affinity for PD-L1. Several studies have used WL12 to construct tracers to assess PD-L1 expression, including [^64^Cu]WL12 145,146, [^68^Ga]WL12 147, and [^18^F]FPy-WL12 148. All of these studies demonstrated the feasibility of WL12 as a tracer ligand involved in PD-L1 imaging. Liu et al. constructed a tracer for the detection of PD-L1 using a small cyclic peptide (SETSKSF) consisting of seven amino acid residues targeting PD-L1, namely ^68^Ga-DOTA-SETSKSF. They indicated that ^68^Ga-DOTA-SETSKSF had high specificity and tumor-to-background ratio **(Fig. 13)**, and that, compared to NOTA-WL12, DOTA-SETSKSF was easier to synthesize 149. In addition, Lv et al. designed a direct [^18^F]FDG-labeled BMS scaffold-based small molecule tracer [^18^F]LG-1 to assess PD-L1 expression in tumors. The [^18^F]LG-1 PET images clearly showed A375-hPD-L1 tumors *in vivo*, and they measured the tumor uptake of this tracer at 60 min to be 3.98 ± 0.21% ID/g. Thus, [^18^F]LG-1 is expected to be a powerful tool for detecting PD-L1 150. Recently, Mishra et al. designed a peptide-based radiotracer, [^68^Ga]Ga-DK223, which binds specifically to PD-L1 with high affinity (K_D_ = 1.01 ± 0.83 nmol/L) and provides high-contrast PET images of PD-L1-expressing mouse tumors within 60 min after administration. The [^68^Ga]Ga-DK223 PET measurement could be used to quantify not only the total PD-L1 levels throughout the body but also the accessible PD-L1 levels—those not occupied by mAbs—during treatment to understand the target engagement of anti-PD-L1 in the tumor; in turn, this could be used to guide immune checkpoint treatment. The results of this study show promise for clinical translation of this radiotracer in humans 151. In addition, Zhou et al. developed a new D-peptide-based tracer (^18^F-NOTA-NF12) and conducted a clinical study. They used this tracer for the first time to image and quantify PD-L1 expression in humans. It was observed that patients with NSCLC and esophageal cancer with high PD-L1 expression had a higher uptake of ^18^F-NOTA-NF12 in their tumors compared to those with low PD-L1 expression. They demonstrated that this tracer is safe for humans and that PET imaging can be completed in less than an hour. However, it should be noted that the uptake of ^18^F-NOTA-NF12 by tumors in animal models and patients is relatively low, which may be related to the insufficient affinity of ^18^F-NOTA-NF12 for PD-L1 (K_D_ = 85.08 nM) 152.

#### 4.4.6. Other

The small non-antibody protein found in the extracellular structural domain of PD-1 has high specificity. Maute et al. inserted a mutation at the “core” position of the PD-1 extracellular domain to acquire a high-affinity ligand, HAC-PD-1, which was coupled to DOTA-maleimide to yield DOTA-HAC and then radiolabeled with ^64^Cu to generate the hPD-L1-specific radio protein ^64^Cu-DOTA-HAC. *In vitro*, ^64^Cu-DOTA-HAC displayed strong immunoreactivity, binding to hPD-L1-positive cells (80.5 ± 1.9%) more readily than to hPD-L1-negative control cells (8.3 ± 0.1%). Moreover, it displayed greater tumor penetration without triggering peripheral effector T cell depletion compared to anti-PD-L1 mAbs, with exceptional benefits 153. In addition, it has been shown that the F(ab′)_2_ fragment has a comparable affinity and specificity to intact antibodies and is cleared more rapidly from the blood 154,155. Cheng et al. obtained the ^124^I-Durva-F(ab′)_2_ tracer by labeling the F(ab′)_2_ fragment of Durvalumab (Durva) with ^124^I. They found that ^124^I-Durva-F(ab′)_2_ had a high affinity for cell lines with high PD-L1 expression (K_D_ = 1.21 ± 0.24 nM). Notably, they quantitatively compared ^124^I-Durva-F(ab′)_2_ with ^124^I-Durva in terms of biodistribution and showed that the peak tumor uptake of ^124^I-Durva-F(ab′)_2_ was similar to that of^ 124^I-Durva but appeared much earlier (5.29 ± 0.42% ID/g for ^124^I-Durva-F(ab′)_2_ at 12 h and 5.18 ± 0.73% ID/g for ^124^I-Durva at 48 h). Furthermore, compared to ^124^I-Durva, ^124^I-Durva-F(ab′)_2_ had faster blood clearance, resulting in a higher tumor-to-background ratio and a higher contrast image. In addition, faster blood clearance of the tracer facilitated a reduction in the radiation dose. Based on the above results, ^124^I-Durva-F(ab′)_2_ is a promising immunoPET tracer for assessing PD-L1 expression in NSCLC xenografts 156.

## 5. Conclusion

In summary, tumor immunity has become a highly researched area in the field of cancer treatment since the discovery of PD-L1. As such, PD-L1 has been studied extensively, and its role in cancer treatment has become increasingly well-understood. It can trigger T cell death, boost the immunosuppressive capacity of Tregs, and regulate and promote EMT, playing a crucial role in tumor immune evasion. Nowadays, PD-L1 is widely used to predict prognosis and guide treatment in clinical settings. The expression of PD-L1 is associated with clinical outcomes, and the difference in PD-L1 expression between primary tumors and metastases can help with biopsy techniques and individualized treatment. Cancer immunotherapies represented by anti-PD-1/PD-L1 antibodies have changed cancer treatment over the past decade. Several anti-PD-1/PD-L1 antibodies have been approved, and numerous PD-L1 mAb combination therapies have been explored in this field. However, the high levels of drug resistance and severe side effects associated with these antibodies have impeded their clinical use, presenting a pressing challenge that must be addressed. To overcome the limits of anti-PD-L1 mAbs, researchers have started to focus on peptide and non-peptide PD-L1 small molecule inhibitors as viable substitutes or supplements to anti-PD-L1 mAbs; however, most of these studies are preclinical, and there is much work to be done before their clinical application. Nevertheless, on a promising note, the combination of a cancer vaccine with PD-L1 blockers is a novel treatment strategy that shows improved OS and PFS and deserves further exploration.

With advances in immunology and molecular biotechnology, quantitative detection of PD-L1 has become an important tool for predicting prognosis and guiding clinical treatment. Traditional immunoassay methods, such as IHC, ELISA, and IF, are still widely used for PD-L1 detection, but novel immunoassays with higher sensitivity, real-time monitoring, and less tumor heterogeneity, such as electrochemical immunoassay, photochemical immunoassay, and nuclear medicine imaging, are emerging trends in this field. With advances in microprocessing technology, modern detection technology is becoming increasingly miniaturized, integrated, and diversified. This will no doubt lead to the development of new, more accurate, and efficient immunoassay techniques for PD-L1 detection, which will be integral to the era of precision cancer therapy.

## Figures and Tables

**Table 1:** The differences in PD-L1 expression between primary tumors and metastases.

Cancer type	Study	Position	N	PD-L1			P	Reference
				positive	negative			
TNBC	RS	primary tumor	179	114(63.7%)		65(36.3%)	P<0.0001	26
		metastasis	161	68(42.2%)		93(57.8%)		
ovarian cancer	RS	primary tumor	194	63(32.5%)		131(67.5%)	P<0.0001	27
		metastasis	194	89(45.9%)		105(54.1%)		
RCC	RS	primary tumor	83	20(24.1%)		63(75.9%)	P=0.44	28
		metastasis	174	50(28.7%)		124(71.3%)		
lung cancer	RS	primary tumor	8285	2356(28.4%)		5929(71.6%)	P<0.0001	29
		metastasis	6743	2276(33.8%)		4467(66.2%)		
bladder carcinoma	RS	primary tumor	142	41(28.9%)		101(71.1%)	NR	30
		metastasis	93	15(16.1%)		78(83.9%)		
								

**Table 2:** Combination treatment strategies for anti-PD-L1 monoclonal antibodies (Take lung cancer, for example).

NCT ID	Combination	Study	N	Primary Endpoint	Reference
NCT03030131	Durvalumab	phase II	46	complete surgical resection rate	52
NCT02904954	Durvalumab+Radiotherapy	phase II	60	MPR	47
NCT03711305	Adebrelimab+Chemotherapy	phase III	462	OS	53
NCT03693300	Durvalumab+Chemoradiotherapy	phase II	117	the incidence of grade 3 or 4 adverse events	54
NCT02542293	Durvalumab+CTLA-4 immunosuppressant	phase III	823	OS	49
NCT03836066	Atezolizumab+Angiogenesis inhibitor	phase II	38	12-month PFS rate	50

**Table 3:** Summary of clinical trials on the PD-L1 small molecule inhibitors.

Inhibitor	NCT ID	Status	Cancer Type	Phase
INCB086550	NCT04629339	Active, not recruiting	NSCLC, UC, RCC, HCC, Melanoma	Phase II
	NCT05101369	Completed	Healthy Volunteers	Phase I
	NCT04674748	Terminated	Solid Tumors	Phase I
	NCT03762447	Active, not recruiting	Solid Tumors	Phase I
GS-4224	NCT04049617	Terminated	Advanced Solid Tumors	Phase I
MAX-10181	NCT05196360	Recruiting	Solid Tumors	Phase I
IMMH-010	NCT04343859	Enrolling by invitation	Malignant Neoplasms	Phase I
ASC61	NCT05287399	Recruiting	Advanced Solid Tumors	Phase I
CA-170	NCT02812875	Completed	Advanced Solid Tumors or Lymphomas	Phase I
BPI-371153	NCT05341557	Not yet recruiting	Advanced Solid Tumors, Lymphoma, NSCLC, HCC	Phase I

## Abbreviations

AgNCs: silver nanoclusters
AuNPs: gold nanoparticles
AuNRs: gold nanorods
CPS: combined positive score
DCs: dendritic cells
ELISA: enzyme-linked immunosorbent assay
EMT: epithelial-mesenchymal transition
EpCAM: epithelial cell adhesion molecule
HCC: hepatocellular carcinoma
IF: immunofluorescence
IHC: immunohistochemistry
LSPR: localized surface plasmon resonance
l-LSPR: longitudinal LSPR
mIF: multiplex immunofluorescence
MNOM: magnetite nanorods containing ordered mesocages
MPR: major pathologic response
NMB: new methylene blue
NR: not reported
NSCLC: non-small cell lung cancer
OS: overall survival
PD-1: programmed death-1
PD-L1: programmed death-ligand 1
PET: positron emission tomography
PFS: progression-free survival
RCC: renal cell carcinoma
RS: retrospective study
SIBP: specific intracellular binding peptide
sPD-1: soluble PD-1
sPD-L1: soluble PD-L1
SPECT: single photon emission computed tomography
SPR: surface plasmon resonance
TILs: tumor-infiltrating lymphocytes
t-LSPR: transverse LSPR
TNBC: triple negative breast cancer
UC: urothelial cancer

## References

[1] Wang X, Teng F, Kong L, Yu J (2016). PD-L1 expression in human cancers and its association with clinical outcomes. Onco Targets Ther.

[2] Ai L, Xu A, Xu J (2020). Roles of PD-1/PD-L1 Pathway: Signaling, Cancer, and Beyond. Adv Exp Med Biol.

[3] Cortez MA, Ivan C, Valdecanas D, Wang X, Peltier HJ, Ye Y (2016). PDL1 Regulation by p53 via miR-34. J Natl Cancer Inst.

[4] Qiu XY, Hu DX, Chen WQ, Chen RQ, Qian SR, Li CY (2018). PD-L1 confers glioblastoma multiforme malignancy via Ras binding and Ras/Erk/EMT activation. Biochim Biophys Acta Mol Basis Dis.

[5] Chen S, Crabill GA, Pritchard TS, McMiller TL, Wei P, Pardoll DM (2019). Mechanisms regulating PD-L1 expression on tumor and immune cells. J Immunother Cancer.

[6] Dong Y, Yang C, Pan F (2021). Post-Translational Regulations of Foxp3 in Treg Cells and Their Therapeutic Applications. Front Immunol.

[7] Cai J, Wang D, Zhang G, Guo X (2019). The Role Of PD-1/PD-L1 Axis In Treg Development And Function: Implications For Cancer Immunotherapy. Onco Targets Ther.

[8] Reynoso ED, Elpek KG, Francisco L, Bronson R, Bellemare-Pelletier A, Sharpe AH (2009). Intestinal tolerance is converted to autoimmune enteritis upon PD-1 ligand blockade. J Immunol.

[9] Chulkina M, Beswick EJ, Pinchuk IV (2020). Role of PD-L1 in Gut Mucosa Tolerance and Chronic Inflammation. Int J Mol Sci.

[10] Gato-Cañas M, Zuazo M, Arasanz H, Ibañez-Vea M, Lorenzo L, Fernandez-Hinojal G (2017). PDL1 Signals through Conserved Sequence Motifs to Overcome Interferon-Mediated Cytotoxicity. Cell Rep.

[11] Cao Y, Liang W, Fang L, Liu MK, Zuo J, Peng YL (2022). PD-L1/PD-L1 signalling promotes colorectal cancer cell migration ability through RAS/MEK/ERK. Clin Exp Pharmacol Physiol.

[12] Quan Z, Yang Y, Zheng H, Zhan Y, Luo J, Ning Y (2022). Clinical implications of the interaction between PD-1/PD-L1 and PI3K/AKT/mTOR pathway in progression and treatment of non-small cell lung cancer. J Cancer.

[13] Yadollahi P, Jeon YK, Ng WL, Choi I (2021). Current understanding of cancer-intrinsic PD-L1: regulation of expression and its protumoral activity. BMB Rep.

[14] Jinesh GG, Manyam GC, Mmeje CO, Baggerly KA, Kamat AM (2017). Surface PD-L1, E-cadherin, CD24, and VEGFR2 as markers of epithelial cancer stem cells associated with rapid tumorigenesis. Sci Rep.

[15] Wu Y, Chen M, Wu P, Chen C, Xu ZP, Gu W (2017). Increased PD-L1 expression in breast and colon cancer stem cells. Clin Exp Pharmacol Physiol.

[16] Gao H, Zhang J, Ren X (2019). PD-L1 regulates tumorigenesis and autophagy of ovarian cancer by activating mTORC signaling. Biosci Rep.

[17] Perrino M, De Vincenzo F, Cordua N, Borea F, Aliprandi M, Santoro A (2023). Immunotherapy with immune checkpoint inhibitors and predictive biomarkers in malignant mesothelioma: Work still in progress. Front Immunol.

[18] Tessier-Cloutier B, Kalloger SE, Al-Kandari M, Milne K, Gao D, Nelson BH (2017). Programmed cell death ligand 1 cut-point is associated with reduced disease specific survival in resected pancreatic ductal adenocarcinoma. BMC Cancer.

[19] Chang B, Huang T, Wei H, Shen L, Zhu D, He W (2019). The correlation and prognostic value of serum levels of soluble programmed death protein 1 (sPD-1) and soluble programmed death-ligand 1 (sPD-L1) in patients with hepatocellular carcinoma. Cancer Immunol Immunother.

[20] Baptista MZ, Sarian LO, Derchain SF, Pinto GA, Vassallo J (2016). Prognostic significance of PD-L1 and PD-L2 in breast cancer. Hum Pathol.

[21] Schalper KA, Velcheti V, Carvajal D, Wimberly H, Brown J, Pusztai L (2014). *In situ* tumor PD-L1 mRNA expression is associated with increased TILs and better outcome in breast carcinomas. Clin Cancer Res.

[22] Huang CY, Chiang SF, Ke TW, Chen TW, You YS, Chen WT (2018). Clinical significance of programmed death 1 ligand-1 (CD274/PD-L1) and intra-tumoral CD8+ T-cell infiltration in stage II-III colorectal cancer. Sci Rep.

[23] Wyss J, Dislich B, Koelzer VH, Galván JA, Dawson H, Hädrich M (2019). Stromal PD-1/PD-L1 Expression Predicts Outcome in Colon Cancer Patients. Clin Colorectal Cancer.

[24] Jiang H, Zhang R, Jiang H, Zhang M, Guo W, Zhang J (2020). Retrospective analysis of the prognostic value of PD-L1 expression and (18)F-FDG PET/CT metabolic parameters in colorectal cancer. J Cancer.

[25] Sun C, Mezzadra R, Schumacher TN (2018). Regulation and Function of the PD-L1 Checkpoint. Immunity.

[26] Rozenblit M, Huang R, Danziger N, Hegde P, Alexander B, Ramkissoon S (2020). Comparison of PD-L1 protein expression between primary tumors and metastatic lesions in triple negative breast cancers. J Immunother Cancer.

[27] Parvathareddy SK, Siraj AK, Al-Badawi IA, Tulbah A, Al-Dayel F, Al-Kuraya KS (2021). Differential expression of PD-L1 between primary and metastatic epithelial ovarian cancer and its clinico-pathological correlation. Sci Rep.

[28] Zhang X, Yin X, Zhang H, Sun G, Yang Y, Chen J (2019). Differential expressions of PD-1, PD-L1 and PD-L2 between primary and metastatic sites in renal cell carcinoma. BMC Cancer.

[29] Moutafi MK, Tao W, Huang R, Haberberger J, Alexander B, Ramkissoon S (2021). Comparison of programmed death-ligand 1 protein expression between primary and metastatic lesions in patients with lung cancer. J Immunother Cancer.

[30] Tretiakova M, Fulton R, Kocherginsky M, Long T, Ussakli C, Antic T (2018). Concordance study of PD-L1 expression in primary and metastatic bladder carcinomas: comparison of four commonly used antibodies and RNA expression. Mod Pathol.

[31] Wang X, Wang F, Zhong M, Yarden Y, Fu L (2020). The biomarkers of hyperprogressive disease in PD-1/PD-L1 blockage therapy. Mol Cancer.

[32] Tang J, Yu JX, Hubbard-Lucey VM, Neftelinov ST, Hodge JP, Lin Y (2018). Trial watch: The clinical trial landscape for PD1/PDL1 immune checkpoint inhibitors. Nat Rev Drug Discov.

[33] Garon EB, Hellmann MD, Rizvi NA, Carcereny E, Leighl NB, Ahn MJ (2019). Five-Year Overall Survival for Patients With Advanced Non-Small-Cell Lung Cancer Treated With Pembrolizumab: Results From the Phase I KEYNOTE-001 Study. J Clin Oncol.

[34] Mahoney KM, Freeman GJ, McDermott DF (2015). The Next Immune-Checkpoint Inhibitors: PD-1/PD-L1 Blockade in Melanoma. Clin Ther.

[35] Rasihashemi SZ, Rezazadeh Gavgani E, Majidazar R, Seraji P, Oladghaffari M, Kazemi T (2022). Tumor-derived exosomal PD-L1 in progression of cancer and immunotherapy. J Cell Physiol.

[36] Ott PA, Bang YJ, Piha-Paul SA, Razak ARA, Bennouna J, Soria JC (2019). T-Cell-Inflamed Gene-Expression Profile, Programmed Death Ligand 1 Expression, and Tumor Mutational Burden Predict Efficacy in Patients Treated With Pembrolizumab Across 20 Cancers: KEYNOTE-028. J Clin Oncol.

[37] Kurz SC, Cabrera LP, Hastie D, Huang R, Unadkat P, Rinne M (2018). PD-1 inhibition has only limited clinical benefit in patients with recurrent high-grade glioma. Neurology.

[38] Wang Z, Wu X (2020). Study and analysis of antitumor resistance mechanism of PD1/PD-L1 immune checkpoint blocker. Cancer Med.

[39] Shi MY, Liu HG, Chen XH, Tian Y, Chen ZN, Wang K (2022). The application basis of immuno-checkpoint inhibitors combined with chemotherapy in cancer treatment. Front Immunol.

[40] Chen X, Zhang W, Yang W, Zhou M, Liu F (2022). Acquired resistance for immune checkpoint inhibitors in cancer immunotherapy: challenges and prospects. Aging (Albany NY).

[41] Li R, Huang B, Tian H, Sun Z (2022). Immune evasion in esophageal squamous cell cancer: From the perspective of tumor microenvironment. Front Oncol.

[42] Loizides S, Constantinidou A (2022). Triple negative breast cancer: Immunogenicity, tumor microenvironment, and immunotherapy. Front Genet.

[43] Hack SP, Zhu AX, Wang Y (2020). Augmenting Anticancer Immunity Through Combined Targeting of Angiogenic and PD-1/PD-L1 Pathways: Challenges and Opportunities. Front Immunol.

[44] Xue Y, Gao S, Gou J, Yin T, He H, Wang Y (2021). Platinum-based chemotherapy in combination with PD-1/PD-L1 inhibitors: preclinical and clinical studies and mechanism of action. Expert Opin Drug Deliv.

[45] Sato H, Okonogi N, Nakano T (2020). Rationale of combination of anti-PD-1/PD-L1 antibody therapy and radiotherapy for cancer treatment. Int J Clin Oncol.

[46] Forde PM, Spicer J, Lu S, Provencio M, Mitsudomi T, Awad MM (2022). Neoadjuvant Nivolumab plus Chemotherapy in Resectable Lung Cancer. N Engl J Med.

[47] Altorki NK, McGraw TE, Borczuk AC, Saxena A, Port JL, Stiles BM (2021). Neoadjuvant durvalumab with or without stereotactic body radiotherapy in patients with early-stage non-small-cell lung cancer: a single-centre, randomised phase 2 trial. Lancet Oncol.

[48] Hicks KC, Fantini M, Donahue RN, Schwab A, Knudson KM, Tritsch SR (2018). Epigenetic priming of both tumor and NK cells augments antibody-dependent cellular cytotoxicity elicited by the anti-PD-L1 antibody avelumab against multiple carcinoma cell types. Oncoimmunology.

[49] de Castro G Jr, Rizvi NA, Schmid P, Syrigos K, Martin C, Yamamoto N (2023). NEPTUNE: Phase 3 Study of First-Line Durvalumab Plus Tremelimumab in Patients With Metastatic NSCLC. J Thorac Oncol.

[50] Provencio M, Ortega AL, Coves-Sarto J, Calvo V, Marsé-Fabregat R, Dómine M (2022). Atezolizumab Plus Bevacizumab as First-line Treatment for Patients With Metastatic Nonsquamous Non-Small Cell Lung Cancer With High Tumor Mutation Burden: A Nonrandomized Controlled Trial. JAMA Oncol.

[51] Upadhaya S, Neftelinov ST, Hodge J, Campbell J (2022). Challenges and opportunities in the PD1/PDL1 inhibitor clinical trial landscape. Nat Rev Drug Discov.

[52] Wislez M, Mazieres J, Lavole A, Zalcman G, Carre O, Egenod T (2022). Neoadjuvant durvalumab for resectable non-small-cell lung cancer (NSCLC): results from a multicenter study (IFCT-1601 IONESCO). J Immunother Cancer.

[53] Wang J, Zhou C, Yao W, Wang Q, Min X, Chen G (2022). Adebrelimab or placebo plus carboplatin and etoposide as first-line treatment for extensive-stage small-cell lung cancer (CAPSTONE-1): a multicentre, randomised, double-blind, placebo-controlled, phase 3 trial. Lancet Oncol.

[54] Garassino MC, Mazieres J, Reck M, Chouaid C, Bischoff H, Reinmuth N (2022). Durvalumab After Sequential Chemoradiotherapy in Stage III, Unresectable NSCLC: The Phase 2 PACIFIC-6 Trial. J Thorac Oncol.

[55] Pan C, Yang H, Lu Y, Hu S, Wu Y, He Q (2021). Recent advance of peptide-based molecules and nonpeptidic small-molecules modulating PD-1/PD-L1 protein-protein interaction or targeting PD-L1 protein degradation. Eur J Med Chem.

[56] Wu Q, Jiang L, Li SC, He QJ, Yang B, Cao J (2021). Small molecule inhibitors targeting the PD-1/PD-L1 signaling pathway. Acta Pharmacol Sin.

[57] Boussiotis VA (2016). Molecular and Biochemical Aspects of the PD-1 Checkpoint Pathway. N Engl J Med.

[58] Akiyama Y, Ashizawa T, Iizuka A, Ando T, Ishikawa Y, Kondou R (2022). Development of Novel Small Antitumor Compounds Inhibiting PD-1/PD-L1 Binding. Anticancer Res.

[59] Guo L, Wei R, Lin Y, Kwok HF (2020). Clinical and Recent Patents Applications of PD-1/PD-L1 Targeting Immunotherapy in Cancer Treatment-Current Progress, Strategy, and Future Perspective. Front Immunol.

[60] Koblish HK, Wu L, Wang LS, Liu PCC, Wynn R, Rios-Doria J (2022). Characterization of INCB086550: A Potent and Novel Small-Molecule PD-L1 Inhibitor. Cancer Discov.

[61] Wu Y, Yang Z, Cheng K, Bi H, Chen J (2022). Small molecule-based immunomodulators for cancer therapy. Acta Pharm Sin B.

[62] Lin X, Lu X, Luo G, Xiang H (2020). Progress in PD-1/PD-L1 pathway inhibitors: From biomacromolecules to small molecules. Eur J Med Chem.

[63] Rekulapelli A, L EF, Iyer G, Balkrishnan R (2023). Effectiveness of immunological agents in non-small cell lung cancer. Cancer Rep (Hoboken).

[64] Malik S, Sah R, Muhammad K, Waheed Y (2023). Tracking HPV Infection, Associated Cancer Development, and Recent Treatment Efforts-A Comprehensive Review. Vaccines (Basel).

[65] Diao K, Chen J, Wu T, Wang X, Wang G, Sun X (2022). Seq2Neo: A Comprehensive Pipeline for Cancer Neoantigen Immunogenicity Prediction. Int J Mol Sci.

[66] Schwarze JK, Geeraerts X, Tuyaerts S, Neyns B (2023). Current "state of the art" on dendritic cell-based cancer vaccines in melanoma. Curr Opin Oncol.

[67] Lim S, Park JH, Chang H (2023). Enhanced anti-tumor immunity of vaccine combined with anti-PD-1 antibody in a murine bladder cancer model. Investig Clin Urol.

[68] Hannani D, Leplus E, Laulagnier K, Chaperot L, Plumas J (2023). Leveraging a powerful allogeneic dendritic cell line towards neoantigen-based cancer vaccines. Genes Cancer.

[69] Guo Z, Yuan Y, Chen C, Lin J, Ma Q, Liu G (2022). Durable complete response to neoantigen-loaded dendritic-cell vaccine following anti-PD-1 therapy in metastatic gastric cancer. NPJ Precis Oncol.

[70] Pan YR, Wu CE, Huang WK, Chen MH, Lan KH, Yeh CN (2022). Chimeric immune checkpoint protein vaccines inhibit the tumorigenesis and growth of rat cholangiocarcinoma. Front Immunol.

[71] Huang KC, Lai CY, Hung WZ, Chang HY, Lin PC, Chiang SF (2023). A Novel Engineered AAV-Based Neoantigen Vaccine in Combination with Radiotherapy Eradicates Tumors. Cancer Immunol Res.

[72] Bai X, Zhou Y, Yokota Y, Matsumoto Y, Zhai B, Maarouf N (2022). Adaptive antitumor immune response stimulated by bio-nanoparticle based vaccine and checkpoint blockade. J Exp Clin Cancer Res.

[73] Xu H, Dong X, Zhao H, Hou T, Chen C, Chen G (2021). Clinical evaluation of a laboratory-developed test using clone E1L3N for the detection of PD-L1 expression status in non-small cell lung cancer. J Clin Lab Anal.

[74] Thunnissen E, de Langen AJ, Smit EF (2017). PD-L1 IHC in NSCLC with a global and methodological perspective. Lung Cancer.

[75] Scheel AH, Dietel M, Heukamp LC, Jöhrens K, Kirchner T, Reu S (2016). Harmonized PD-L1 immunohistochemistry for pulmonary squamous-cell and adenocarcinomas. Mod Pathol.

[76] Hirsch FR, McElhinny A, Stanforth D, Ranger-Moore J, Jansson M, Kulangara K (2017). PD-L1 Immunohistochemistry Assays for Lung Cancer: Results from Phase 1 of the Blueprint PD-L1 IHC Assay Comparison Project. J Thorac Oncol.

[77] Ratcliffe MJ, Sharpe A, Midha A, Barker C, Scott M, Scorer P (2017). Agreement between Programmed Cell Death Ligand-1 Diagnostic Assays across Multiple Protein Expression Cutoffs in Non-Small Cell Lung Cancer. Clin Cancer Res.

[78] Ilie M, Long-Mira E, Bence C, Butori C, Lassalle S, Bouhlel L (2016). Comparative study of the PD-L1 status between surgically resected specimens and matched biopsies of NSCLC patients reveal major discordances: a potential issue for anti-PD-L1 therapeutic strategies. Ann Oncol.

[79] Takeda M, Kasai T, Naito M, Tamiya A, Taniguchi Y, Saijo N (2019). Programmed Death-ligand 1 Expression With Clone 22C3 in Non-small Cell Lung Cancer: A Single Institution Experience. Clin Med Insights Oncol.

[80] Herbst RS, Baas P, Kim DW, Felip E, Pérez-Gracia JL, Han JY (2016). Pembrolizumab versus docetaxel for previously treated, PD-L1-positive, advanced non-small-cell lung cancer (KEYNOTE-010): a randomised controlled trial. Lancet.

[81] Spigel DR, Chaft JE, Gettinger S, Chao BH, Dirix L, Schmid P (2018). FIR: Efficacy, Safety, and Biomarker Analysis of a Phase II Open-Label Study of Atezolizumab in PD-L1-Selected Patients With NSCLC. J Thorac Oncol.

[82] Jeong S, Lee N, Park MJ, Jeon K, Song W (2021). Currently Used Laboratory Methodologies for Assays Detecting PD-1, PD-L1, PD-L2 and Soluble PD-L1 in Patients with Metastatic Breast Cancer. Cancers (Basel).

[83] Huang M, Yang J, Wang T, Song J, Xia J, Wu L (2020). Homogeneous, Low-volume, Efficient, and Sensitive Quantitation of Circulating Exosomal PD-L1 for Cancer Diagnosis and Immunotherapy Response Prediction. Angew Chem Int Ed Engl.

[84] Zhand S, Razmjou A, Azadi S, Bazaz SR, Shrestha J, Jahromi MAF (2020). Metal-Organic Framework-Enhanced ELISA Platform for Ultrasensitive Detection of PD-L1. ACS Appl Bio Mater.

[85] Takeuchi M, Doi T, Obayashi K, Hirai A, Yoneda K, Tanaka F (2018). Soluble PD-L1 with PD-1-binding capacity exists in the plasma of patients with non-small cell lung cancer. Immunol Lett.

[86] Jeong S, Park MJ, Song W, Kim HS (2020). Current immunoassay methods and their applications to clinically used biomarkers of breast cancer. Clin Biochem.

[87] Yeong J, Tan T, Chow ZL, Cheng Q, Lee B, Seet A (2020). Multiplex immunohistochemistry/immunofluorescence (mIHC/IF) for PD-L1 testing in triple-negative breast cancer: a translational assay compared with conventional IHC. J Clin Pathol.

[88] Yaseen Z, Gide TN, Conway JW, Potter AJ, Quek C, Hong AM (2022). Validation of an Accurate Automated Multiplex Immunofluorescence Method for Immuno-Profiling Melanoma. Front Mol Biosci.

[89] Vahadane A, Sharma S, Mandal D, Dabbeeru M, Jakthong J, Garcia-Guzman M (2023). Development of an automated combined positive score prediction pipeline using artificial intelligence on multiplexed immunofluorescence images. Comput Biol Med.

[90] Xing Y, Liu J, Sun S, Ming T, Wang Y, Luo J (2021). New electrochemical method for programmed death-ligand 1 detection based on a paper-based microfluidic aptasensor. Bioelectrochemistry.

[91] Wang Y, Zhao G, Zhang G, Zhang Y, Wang H, Cao W (2020). An electrochemical aptasensor based on gold-modified MoS2/rGO nanocomposite and gold-palladium-modified Fe-MOFs for sensitive detection of lead ions. Sensor Actuat B-Chem.

[92] Wu Y, Tilley RD, Gooding JJ (2019). Challenges and Solutions in Developing Ultrasensitive Biosensors. J Am Chem Soc.

[93] Zhao J, Zhang P, Wang J, Xi Q, Zhao X, Ji M (2017). Plasma levels of soluble programmed death ligand-1 may be associated with overall survival in nonsmall cell lung cancer patients receiving thoracic radiotherapy. Medicine (Baltimore).

[94] Xing Y, Liu J, Luo J, Ming T, Yang G, Sun S (2022). A Dual-Channel Intelligent Point-of-Care Testing System for Soluble Programmed Death-1 and Programmed Death-Ligand 1 Detection Based on Folding Paper-Based Immunosensors. ACS Sens.

[95] Moazzam P, Myekhlai M, Alinezhad A, Alshawawreh FA, Bakthavathsalam P, Gonçales VR (2021). Ultrasensitive detection of programmed death-ligand 1 (PD-L1) in whole blood using dispersible electrodes. Chem Commun (Camb).

[96] Gong L, Feng L, Zheng Y, Luo Y, Zhu D, Chao J (2022). Molybdenum Disulfide-Based Nanoprobes: Preparation and Sensing Application. Biosensors (Basel).

[97] Du X, Li Y, Zhang Z, Zhang C, Hu J, Wang X (2022). An electrochemical biosensor for the assessment of tumor immunotherapy based on the detection of immune checkpoint protein programmed death ligand-1. Biosens Bioelectron.

[98] Mao Z, Zhu H, Peng X, Chen J, Chen Q, Chen X (2022). *In situ* vertical alignment of 2D MoS(2) layers on GO film: enhanced electrochemical properties for PD-L1 sensing. Mikrochim Acta.

[99] Jiang Y, Zhu P, Zhao J, Li S, Wu Y, Xiong X (2023). Sensitive biosensors based on topological insulator Bi(2)Se(3) and peptide. Anal Chim Acta.

[100] Luo C, Xin H, Zhou Z, Hu Z, Sun R, Yao N (2022). Tumor-derived exosomes induce immunosuppressive macrophages to foster intrahepatic cholangiocarcinoma progression. Hepatology.

[101] Xie F, Xu M, Lu J, Mao L, Wang S (2019). The role of exosomal PD-L1 in tumor progression and immunotherapy. Mol Cancer.

[102] Chen G, Huang AC, Zhang W, Zhang G, Wu M, Xu W (2018). Exosomal PD-L1 contributes to immunosuppression and is associated with anti-PD-1 response. Nature.

[103] Sha L, Bo B, Yang F, Li J, Cao Y, Zhao J (2022). Programmable DNA-Fueled Electrochemical Analysis of Lung Cancer Exosomes. Anal Chem.

[104] Maccaferri N, Zhao Y, Isoniemi T, Iarossi M, Parracino A, Strangi G (2019). Hyperbolic Meta-Antennas Enable Full Control of Scattering and Absorption of Light. Nano Lett.

[105] Han H, Hou D, Zhao L, Luan N, Song L, Liu Z (2020). A Large Detection-Range Plasmonic Sensor Based on An H-Shaped Photonic Crystal Fiber. Sensors (Basel).

[106] Niu M, Liu Y, Yi M, Jiao D, Wu K (2022). Biological Characteristics and Clinical Significance of Soluble PD-1/PD-L1 and Exosomal PD-L1 in Cancer. Front Immunol.

[107] Huang X, Zhang ZH, Chen J, Mao Z, Zhu H, Liu Y (2021). One dimensional magneto-optical nanocomplex from silver nanoclusters and magnetite nanorods containing ordered mesocages for sensitive detection of PD-L1. Biosens Bioelectron.

[108] Bailly C, Thuru X, Quesnel B (2021). Soluble Programmed Death Ligand-1 (sPD-L1): A Pool of Circulating Proteins Implicated in Health and Diseases. Cancers (Basel).

[109] Hu J, Zhang ZH, Zhu Z, Chen J, Hu X, Chen H (2021). Specific intracellular binding peptide as sPD-L1 antibody mimic: Robust binding capacity and intracellular region specific modulation upon applied to sensing research. Biosens Bioelectron.

[110] Cordonnier M, Nardin C, Chanteloup G, Derangere V, Algros MP, Arnould L (2020). Tracking the evolution of circulating exosomal-PD-L1 to monitor melanoma patients. J Extracell Vesicles.

[111] Liu C, Zeng X, An Z, Yang Y, Eisenbaum M, Gu X (2018). Sensitive Detection of Exosomal Proteins via a Compact Surface Plasmon Resonance Biosensor for Cancer Diagnosis. ACS Sens.

[112] Zhang J, Zhu Y, Guan M, Liu Y, Lv M, Zhang C (2022). Isolation of circulating exosomes and identification of exosomal PD-L1 for predicting immunotherapy response. Nanoscale.

[113] Wang Y, Mao Z, Chen Q, Koh K, Hu X, Chen H (2022). Rapid and sensitive detection of PD-L1 exosomes using Cu-TCPP 2D MOF as a SPR sensitizer. Biosens Bioelectron.

[114] He L, He F, Feng Y, Wang X, Li Y, Tian Y (2021). Hybridized nanolayer modified Omega-shaped fiber-optic synergistically enhances localized surface plasma resonance for ultrasensitive cytosensor and efficient photothermal therapy. Biosens Bioelectron.

[115] Xie L, Yan X, Du Y (2014). An aptamer based wall-less LSPR array chip for label-free and high throughput detection of biomolecules. Biosens Bioelectron.

[116] Takemura K (2021). Surface Plasmon Resonance (SPR)- and Localized SPR (LSPR)-Based Virus Sensing Systems: Optical Vibration of Nano- and Micro-Metallic Materials for the Development of Next-Generation Virus Detection Technology. Biosensors (Basel).

[117] Li J, Wang H, Li Z, Su Z, Zhu Y (2020). Preparation and Application of Metal Nanoparticals Elaborated Fiber Sensors. Sensors (Basel).

[118] Pellas V, Hu D, Mazouzi Y, Mimoun Y, Blanchard J, Guibert C (2020). Gold Nanorods for LSPR Biosensing: Synthesis, Coating by Silica, and Bioanalytical Applications. Biosensors (Basel).

[119] Wang C, Huang CH, Gao Z, Shen J, He J, MacLachlan A (2021). Nanoplasmonic Sandwich Immunoassay for Tumor-Derived Exosome Detection and Exosomal PD-L1 Profiling. ACS Sens.

[120] Luo B, Wang Y, Lu H, Wu S, Lu Y, Shi S (2019). Label-free and specific detection of soluble programmed death ligand-1 using a localized surface plasmon resonance biosensor based on excessively tilted fiber gratings. Biomed Opt Express.

[121] Lv G, Sun X, Qiu L, Sun Y, Li K, Liu Q (2020). PET Imaging of Tumor PD-L1 Expression with a Highly Specific Nonblocking Single-Domain Antibody. J Nucl Med.

[122] Wang W, Gao Z, Wang L, Li J, Yu J, Han S (2020). Application and Prospects of Molecular Imaging in Immunotherapy. Cancer Manag Res.

[123] Natarajan A, Mayer AT, Xu L, Reeves RE, Gano J, Gambhir SS (2015). Novel Radiotracer for ImmunoPET Imaging of PD-1 Checkpoint Expression on Tumor Infiltrating Lymphocytes. Bioconjug Chem.

[124] Heskamp S, Hobo W, Molkenboer-Kuenen JD, Olive D, Oyen WJ, Dolstra H (2015). Noninvasive Imaging of Tumor PD-L1 Expression Using Radiolabeled Anti-PD-L1 Antibodies. Cancer Res.

[125] Bensch F, van der Veen EL, Lub-de Hooge MN, Jorritsma-Smit A, Boellaard R, Kok IC (2018). (89)Zr-atezolizumab imaging as a non-invasive approach to assess clinical response to PD-L1 blockade in cancer. Nat Med.

[126] Jagoda EM, Vasalatiy O, Basuli F, Opina ACL, Williams MR, Wong K (2019). Immuno-PET Imaging of the Programmed Cell Death-1 Ligand (PD-L1) Using a Zirconium-89 Labeled Therapeutic Antibody, Avelumab. Mol Imaging.

[127] Christensen C, Kristensen LK, Alfsen MZ, Nielsen CH, Kjaer A (2020). Quantitative PET imaging of PD-L1 expression in xenograft and syngeneic tumour models using a site-specifically labelled PD-L1 antibody. Eur J Nucl Med Mol Imaging.

[128] Krache A, Fontan C, Pestourie C, Bardiès M, Bouvet Y, Payoux P (2021). Preclinical Pharmacokinetics and Dosimetry of an (89)Zr Labelled Anti-PDL1 in an Orthotopic Lung Cancer Murine Model. Front Med (Lausanne).

[129] Li D, Wang F, Jiang J, Hou X, Ding J, Wang Z (2022). Construction of an Iodine-Labeled CS1001 Antibody for Targeting PD-L1 Detection and Comparison with Low-Molecular-Peptide Micro-PET Imaging. Mol Pharm.

[130] Lecocq Q, De Vlaeminck Y, Hanssens H, D'Huyvetter M, Raes G, Goyvaerts C (2019). Theranostics in immuno-oncology using nanobody derivatives. Theranostics.

[131] Broos K, Lecocq Q, Xavier C, Bridoux J, Nguyen TT, Corthals J (2019). Evaluating a Single Domain Antibody Targeting Human PD-L1 as a Nuclear Imaging and Therapeutic Agent. Cancers (Basel).

[132] Xing Y, Chand G, Liu C, Cook GJR, O'Doherty J, Zhao L (2019). Early Phase I Study of a (99m)Tc-Labeled Anti-Programmed Death Ligand-1 (PD-L1) Single-Domain Antibody in SPECT/CT Assessment of PD-L1 Expression in Non-Small Cell Lung Cancer. J Nucl Med.

[133] Zhang Y, Ding Y, Li N, Wang S, Zhou S, Li R (2023). Noninvasive Imaging of Tumor PD-L1 Expression Using [(99m)Tc]Tc-Labeled KN035 with SPECT/CT. Mol Pharm.

[134] Liu Q, Jiang L, Li K, Li H, Lv G, Lin J (2021). Immuno-PET imaging of (68)Ga-labeled nanobody Nb109 for dynamic monitoring the PD-L1 expression in cancers. Cancer Immunol Immunother.

[135] Chen Y, Zhu S, Fu J, Lin J, Sun Y, Lv G (2022). Development of a radiolabeled site-specific single-domain antibody positron emission tomography probe for monitoring PD-L1 expression in cancer. J Pharm Anal.

[136] Lipovsek D (2011). Adnectins: engineered target-binding protein therapeutics. Protein Eng Des Sel.

[137] Donnelly DJ, Smith RA, Morin P, Lipovšek D, Gokemeijer J, Cohen D (2018). Synthesis and Biologic Evaluation of a Novel (18)F-Labeled Adnectin as a PET Radioligand for Imaging PD-L1 Expression. J Nucl Med.

[138] Nienhuis PH, Antunes IF, Glaudemans A, Jalving M, Leung D, Noordzij W (2022). (18)F-BMS986192 PET Imaging of PD-L1 in Metastatic Melanoma Patients with Brain Metastases Treated with Immune Checkpoint Inhibitors: A Pilot Study. J Nucl Med.

[139] Robu S, Richter A, Gosmann D, Seidl C, Leung D, Hayes W (2021). Synthesis and Preclinical Evaluation of a (68)Ga-Labeled Adnectin, (68)Ga-BMS-986192, as a PET Agent for Imaging PD-L1 Expression. J Nucl Med.

[140] Zhou H, Bao G, Wang Z, Zhang B, Li D, Chen L (2022). PET imaging of an optimized anti-PD-L1 probe (68)Ga-NODAGA-BMS986192 in immunocompetent mice and non-human primates. EJNMMI Res.

[141] Löfblom J, Feldwisch J, Tolmachev V, Carlsson J, Ståhl S, Frejd FY (2010). Affibody molecules: engineered proteins for therapeutic, diagnostic and biotechnological applications. FEBS Lett.

[142] González Trotter DE, Meng X, McQuade P, Rubins D, Klimas M, Zeng Z (2017). *In vivo* Imaging of the Programmed Death Ligand 1 by (18)F PET. J Nucl Med.

[143] Rubins DJ, Meng X, McQuade P, Klimas M, Getty K, Lin SA (2021). *In vivo* Evaluation and Dosimetry Estimate for a High Affinity Affibody PET Tracer Targeting PD-L1. Mol Imaging Biol.

[144] Liang Z, Hu X, Hu H, Wang P, Cai J (2022). Novel small (99m)Tc-labeled affibody molecular probe for PD-L1 receptor imaging. Front Oncol.

[145] Chatterjee S, Lesniak WG, Miller MS, Lisok A, Sikorska E, Wharram B (2017). Rapid PD-L1 detection in tumors with PET using a highly specific peptide. Biochem Biophys Res Commun.

[146] Kumar D, Lisok A, Dahmane E, McCoy M, Shelake S, Chatterjee S (2019). Peptide-based PET quantifies target engagement of PD-L1 therapeutics. J Clin Invest.

[147] De Silva RA, Kumar D, Lisok A, Chatterjee S, Wharram B, Venkateswara Rao K (2018). Peptide-Based (68)Ga-PET Radiotracer for Imaging PD-L1 Expression in Cancer. Mol Pharm.

[148] Lesniak WG, Mease RC, Chatterjee S, Kumar D, Lisok A, Wharram B (2019). Development of [(18)F]FPy-WL12 as a PD-L1 Specific PET Imaging Peptide. Mol Imaging.

[149] Liu H, Hu M, Deng J, Zhao Y, Peng D, Feng Y (2022). A Novel Small Cyclic Peptide-Based (68)Ga-Radiotracer for Positron Emission Tomography Imaging of PD-L1 Expression in Tumors. Mol Pharm.

[150] Lv G, Miao Y, Chen Y, Lu C, Wang X, Xie M (2021). Promising potential of a (18)F-labelled small-molecular radiotracer to evaluate PD-L1 expression in tumors by PET imaging. Bioorg Chem.

[151] Mishra A, Kumar D, Gupta K, Lofland G, Sharma AK, Banka DS (2023). Gallium-68-labeled Peptide PET Quantifies Tumor Exposure of PD-L1 Therapeutics. Clin Cancer Res.

[152] Zhou M, Wang X, Chen B, Xiang S, Rao W, Zhang Z (2022). Preclinical and first-in-human evaluation of (18)F-labeled D-peptide antagonist for PD-L1 status imaging with PET. Eur J Nucl Med Mol Imaging.

[153] Maute RL, Gordon SR, Mayer AT, McCracken MN, Natarajan A, Ring NG (2015). Engineering high-affinity PD-1 variants for optimized immunotherapy and immuno-PET imaging. Proc Natl Acad Sci U S A.

[154] Spicer CD, Jumeaux C, Gupta B, Stevens MM (2018). Peptide and protein nanoparticle conjugates: versatile platforms for biomedical applications. Chem Soc Rev.

[155] Sham JG, Kievit FM, Grierson JR, Chiarelli PA, Miyaoka RS, Zhang M (2014). Glypican-3-targeting F(ab')2 for 89Zr PET of hepatocellular carcinoma. J Nucl Med.

[156] Cheng Y, Shi D, Xu Z, Gao Z, Si Z, Zhao Y (2022). (124)I-Labeled Monoclonal Antibody and Fragment for the Noninvasive Evaluation of Tumor PD-L1 Expression *In vivo*. Mol Pharm.

